# Supplementary figures and images for: Computational design of novel Cas9 PAM-interacting domains using evolution-based modelling and structural quality assessment
Source: PLoS Comput Biol. 2023 Nov 17;19(11):e1011621. doi: 10.1371/journal.pcbi.1011621 (PMC10729993; doi:10.1371/journal.pcbi.1011621)

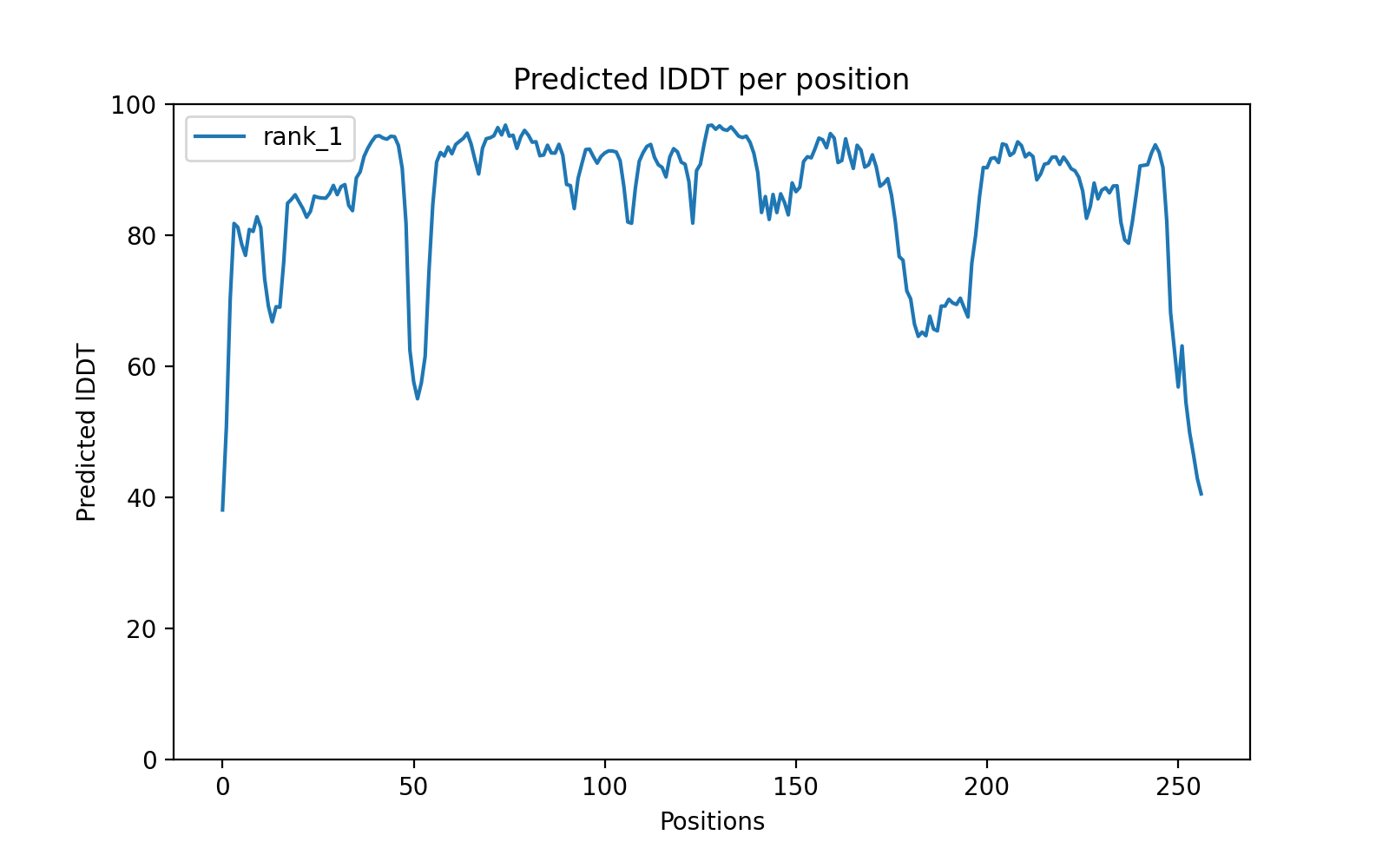

Supplement: S1 File — This file contains the experimentally tested sequences in a zip format. It includes Fasta files, AlphaFold Prediction with PDB file, pLDDT, and more. (ZIP) [file pcbi.1011621.s001.zip › plddt/dg_10_plddt.png]

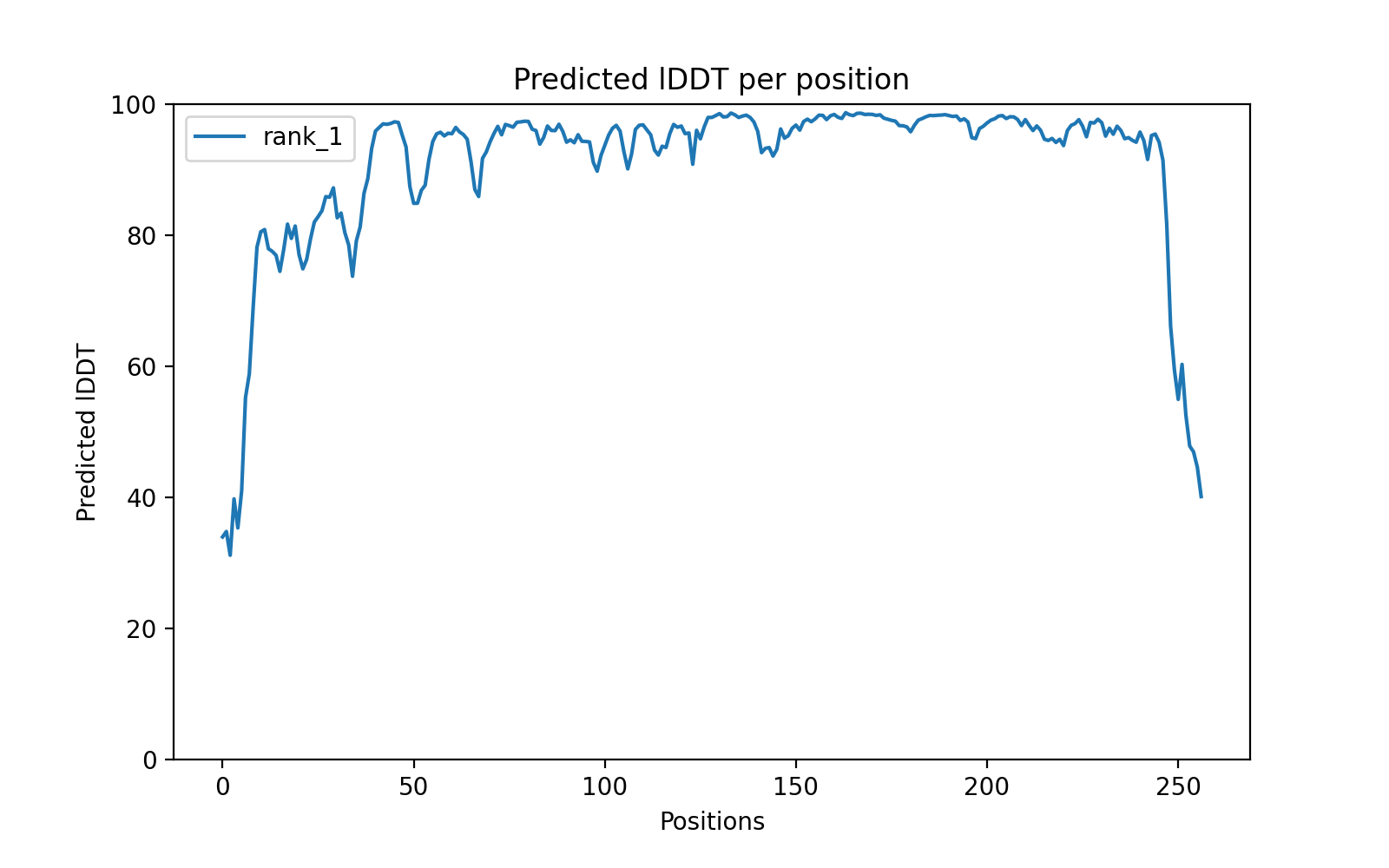

Supplement: S1 File — This file contains the experimentally tested sequences in a zip format. It includes Fasta files, AlphaFold Prediction with PDB file, pLDDT, and more. (ZIP) [file pcbi.1011621.s001.zip › plddt/dgfx_15_plddt.png]

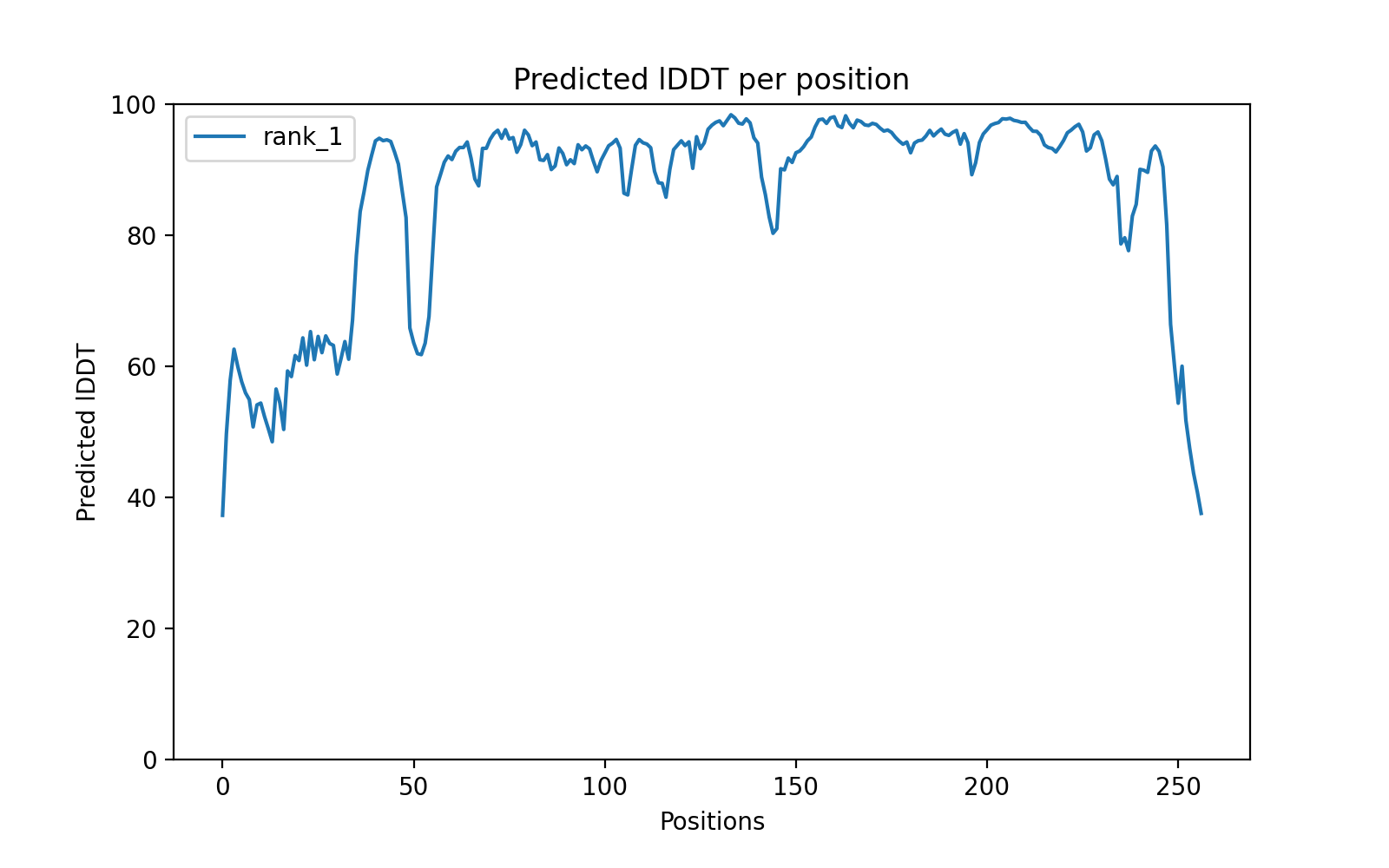

Supplement: S1 File — This file contains the experimentally tested sequences in a zip format. It includes Fasta files, AlphaFold Prediction with PDB file, pLDDT, and more. (ZIP) [file pcbi.1011621.s001.zip › plddt/dg_3_plddt.png]

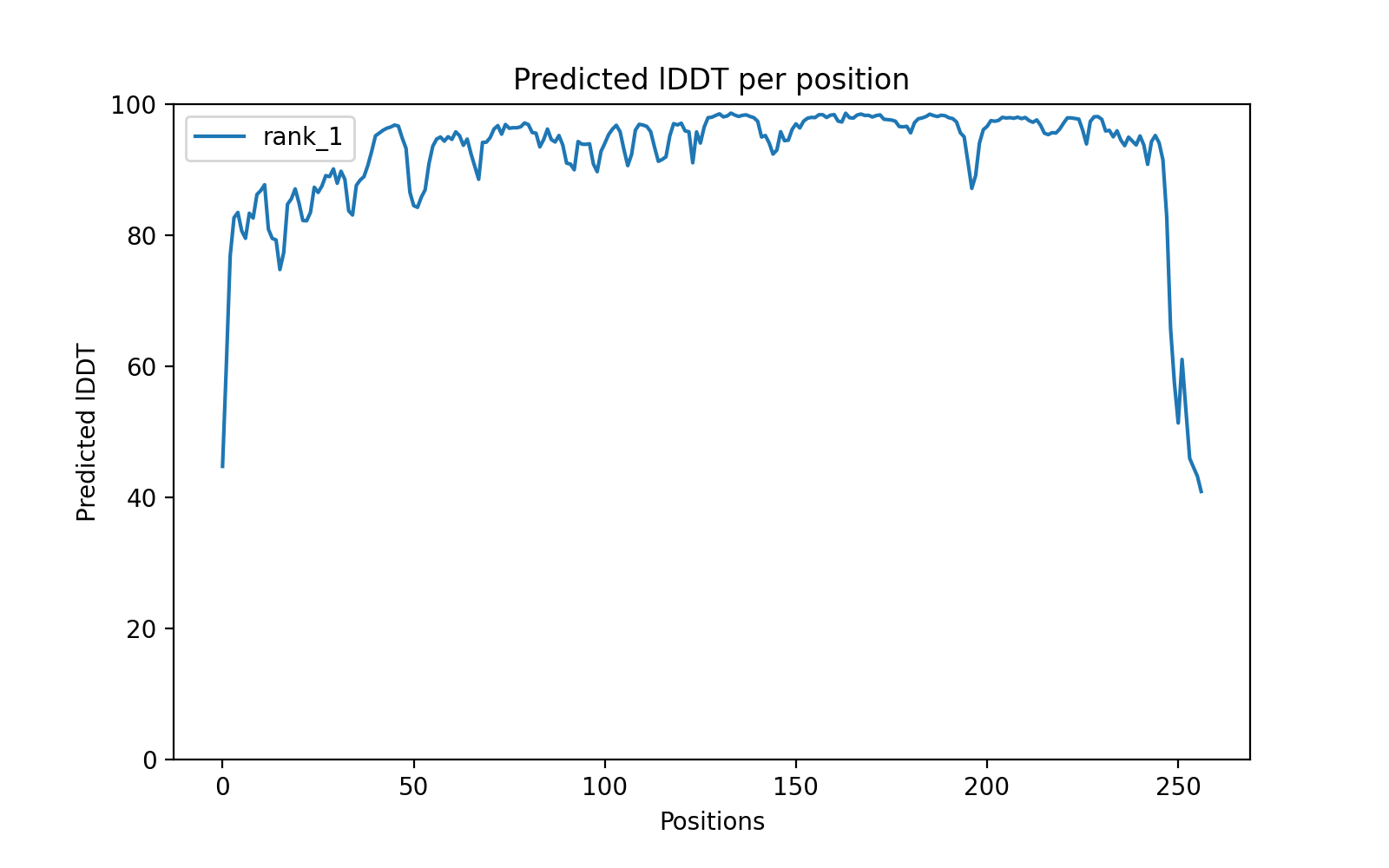

Supplement: S1 File — This file contains the experimentally tested sequences in a zip format. It includes Fasta files, AlphaFold Prediction with PDB file, pLDDT, and more. (ZIP) [file pcbi.1011621.s001.zip › plddt/dgfx_69_plddt.png]

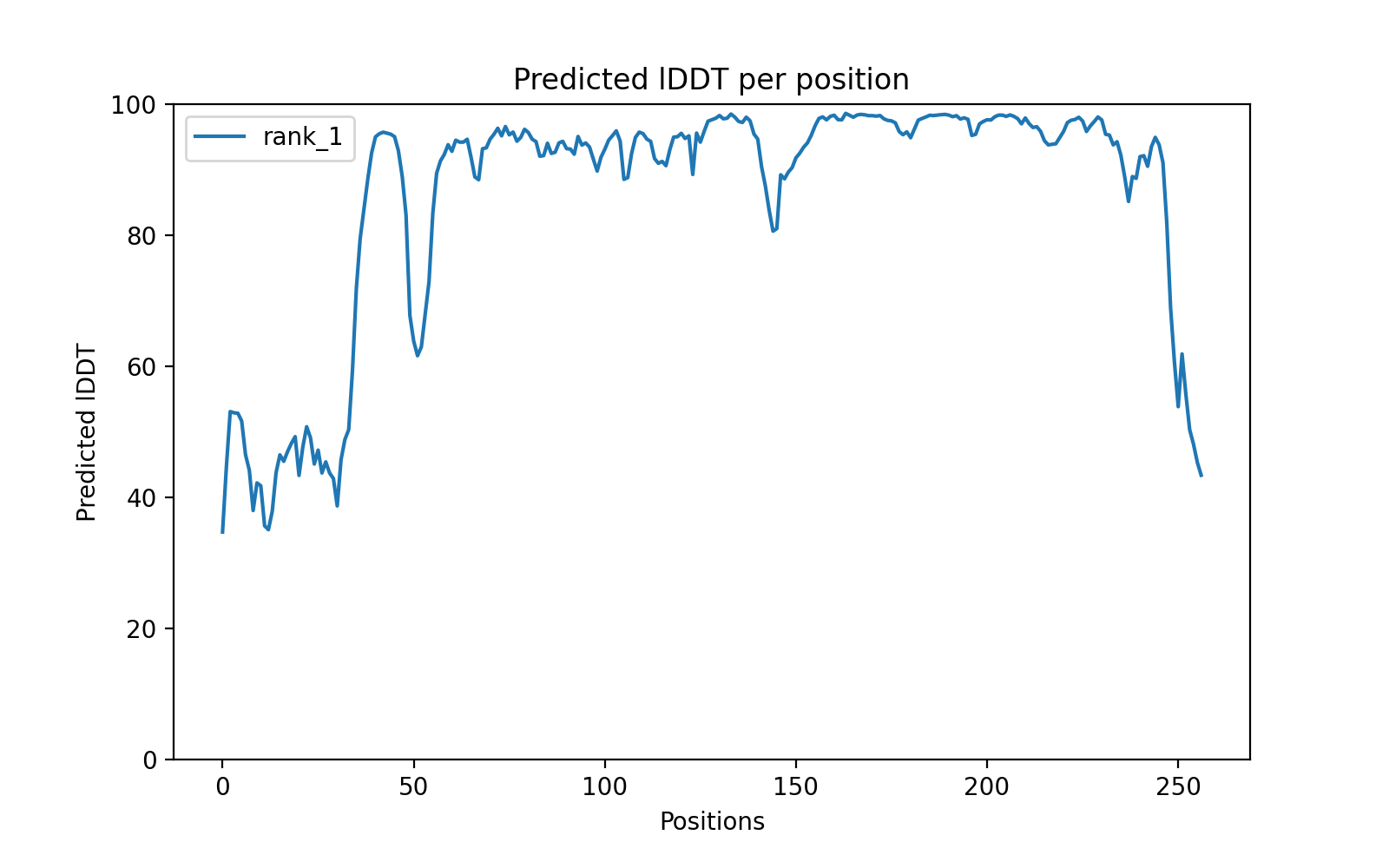

Supplement: S1 File — This file contains the experimentally tested sequences in a zip format. It includes Fasta files, AlphaFold Prediction with PDB file, pLDDT, and more. (ZIP) [file pcbi.1011621.s001.zip › plddt/dg_1_plddt.png]

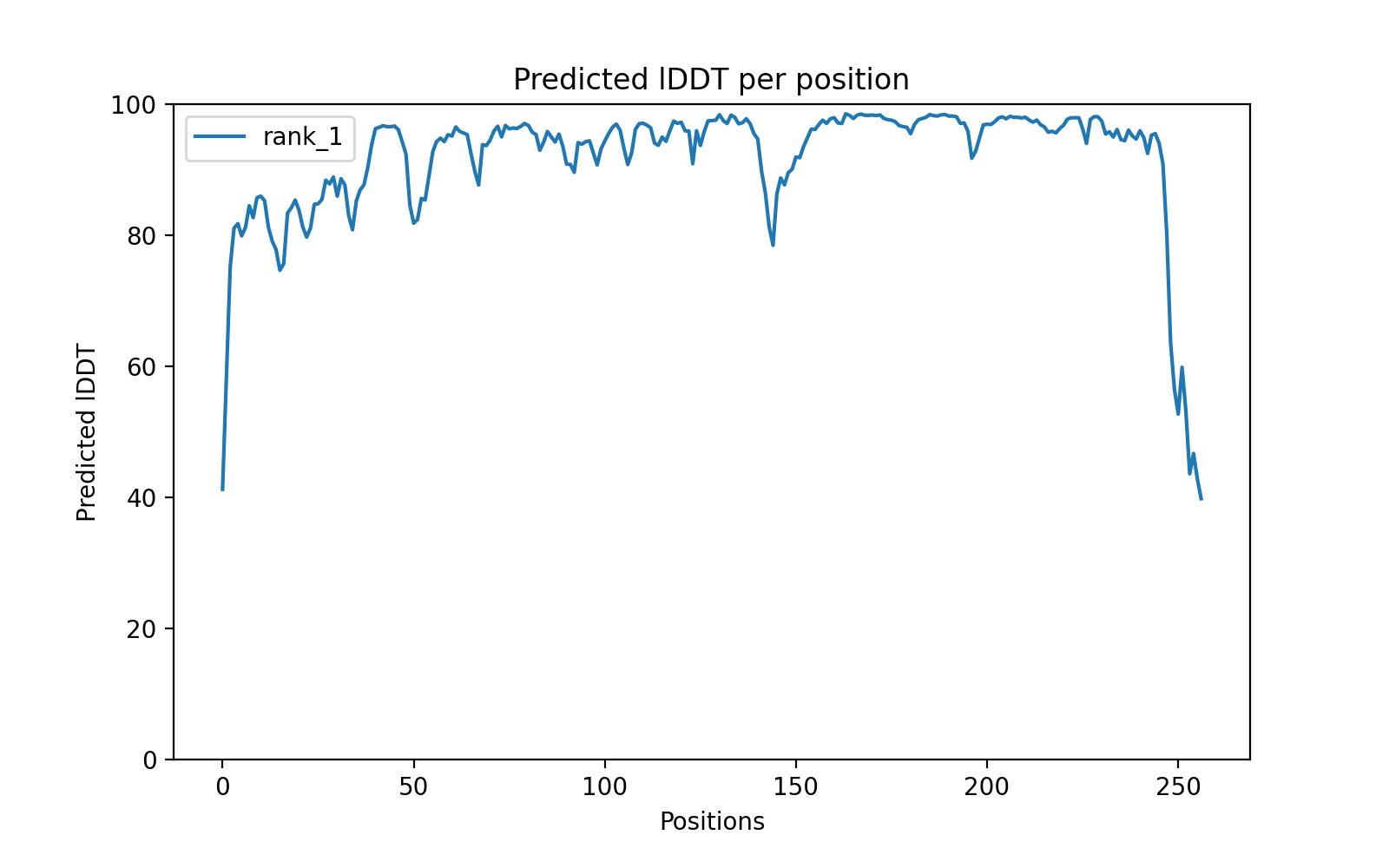

Supplement: S1 File — This file contains the experimentally tested sequences in a zip format. It includes Fasta files, AlphaFold Prediction with PDB file, pLDDT, and more. (ZIP) [file pcbi.1011621.s001.zip › plddt/dgfx_34_plddt.png]

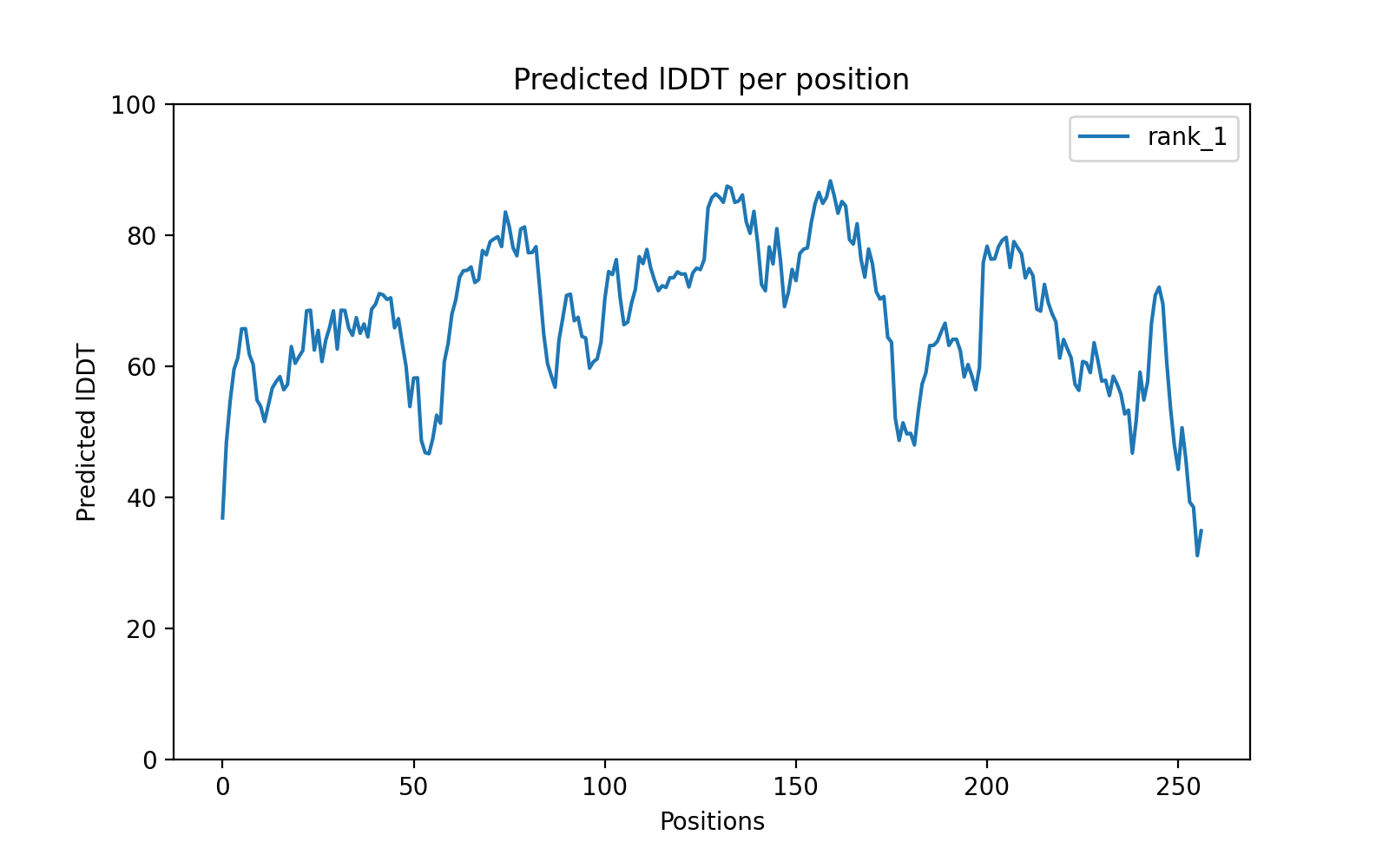

Supplement: S1 File — This file contains the experimentally tested sequences in a zip format. It includes Fasta files, AlphaFold Prediction with PDB file, pLDDT, and more. (ZIP) [file pcbi.1011621.s001.zip › plddt/dg_22_plddt.png]

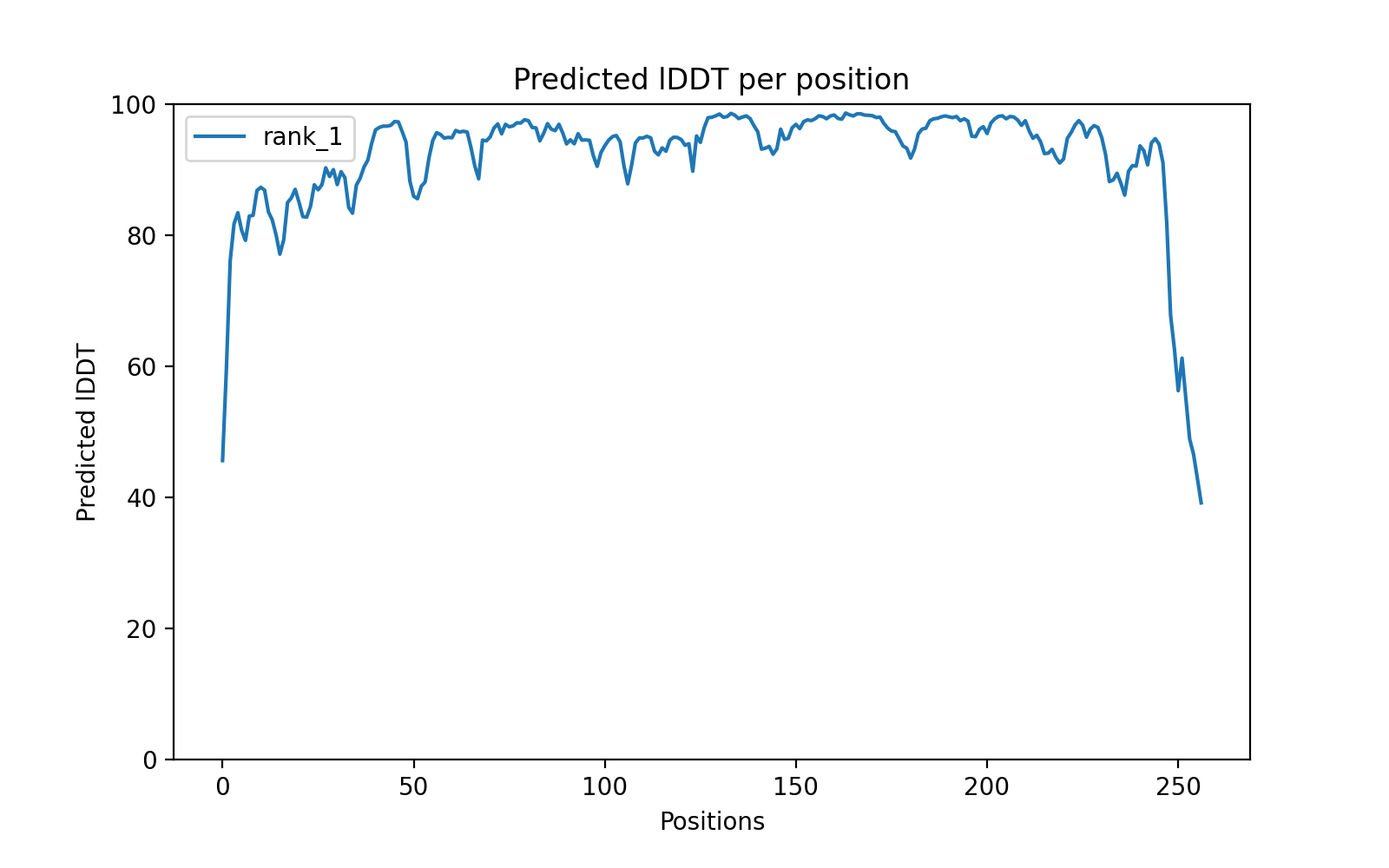

Supplement: S1 File — This file contains the experimentally tested sequences in a zip format. It includes Fasta files, AlphaFold Prediction with PDB file, pLDDT, and more. (ZIP) [file pcbi.1011621.s001.zip › plddt/dgfx_22_plddt.png]

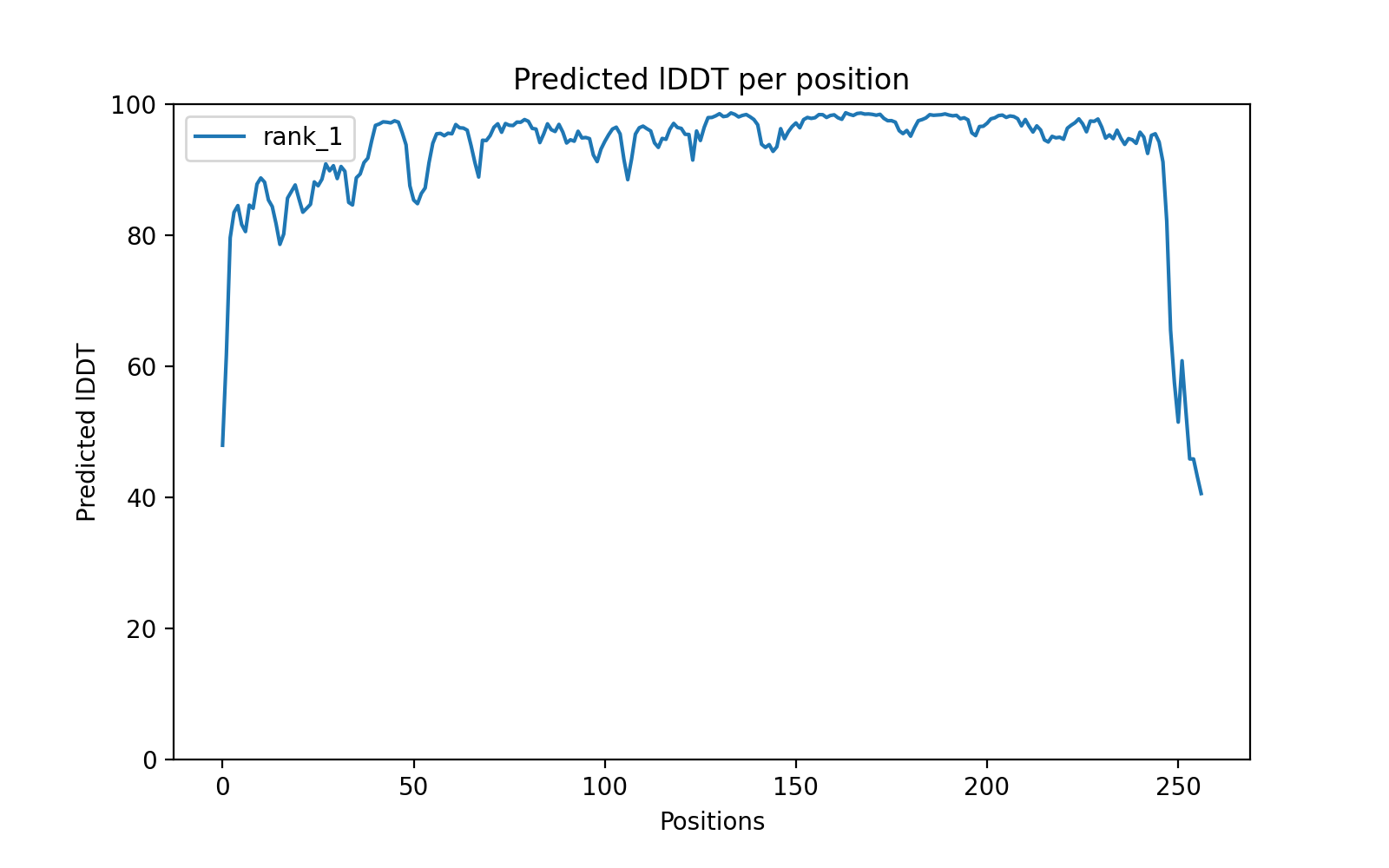

Supplement: S1 File — This file contains the experimentally tested sequences in a zip format. It includes Fasta files, AlphaFold Prediction with PDB file, pLDDT, and more. (ZIP) [file pcbi.1011621.s001.zip › plddt/dgfx_7_plddt.png]

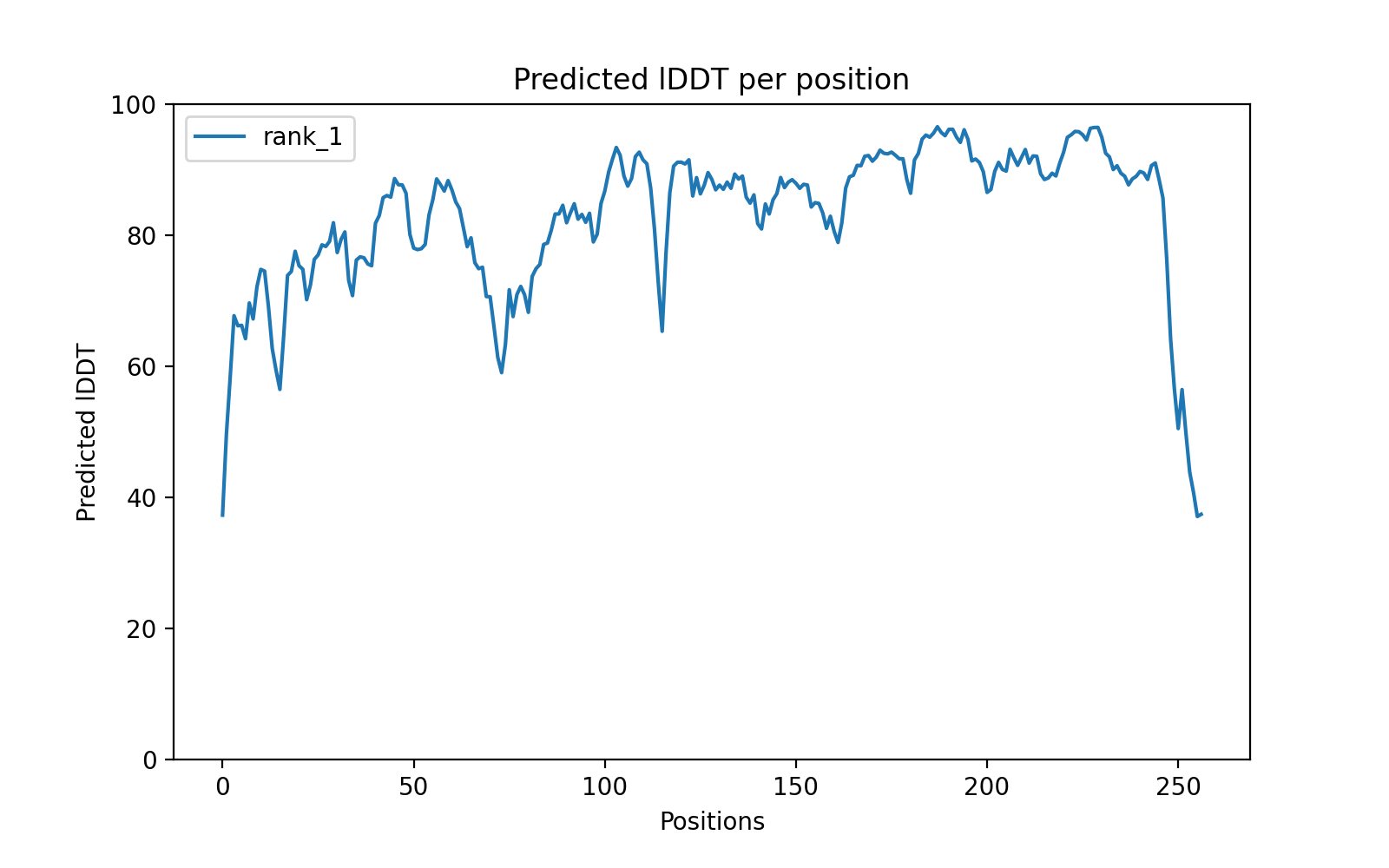

Supplement: S1 File — This file contains the experimentally tested sequences in a zip format. It includes Fasta files, AlphaFold Prediction with PDB file, pLDDT, and more. (ZIP) [file pcbi.1011621.s001.zip › plddt/dg_15_plddt.png]

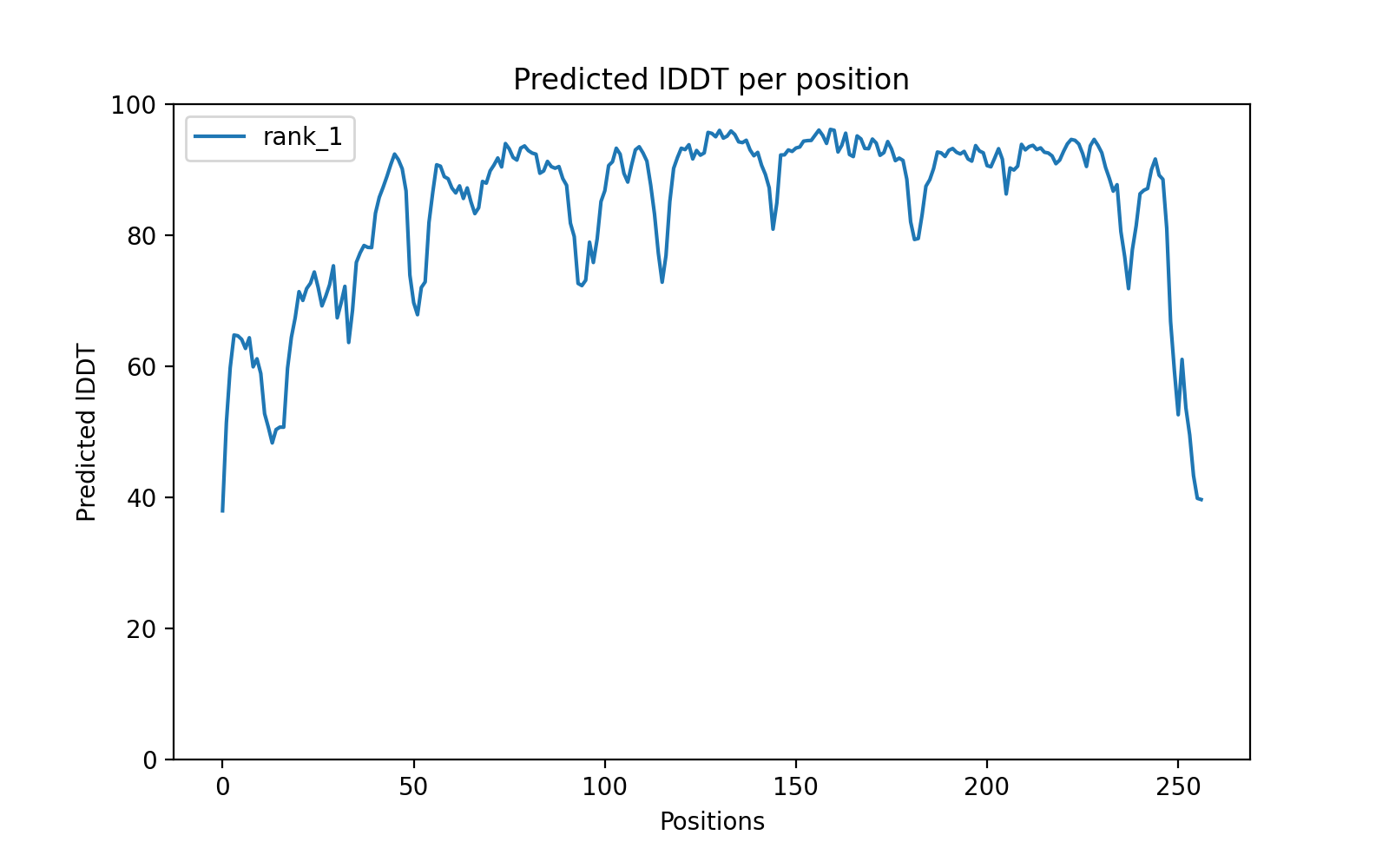

Supplement: S1 File — This file contains the experimentally tested sequences in a zip format. It includes Fasta files, AlphaFold Prediction with PDB file, pLDDT, and more. (ZIP) [file pcbi.1011621.s001.zip › plddt/dg_9_plddt.png]

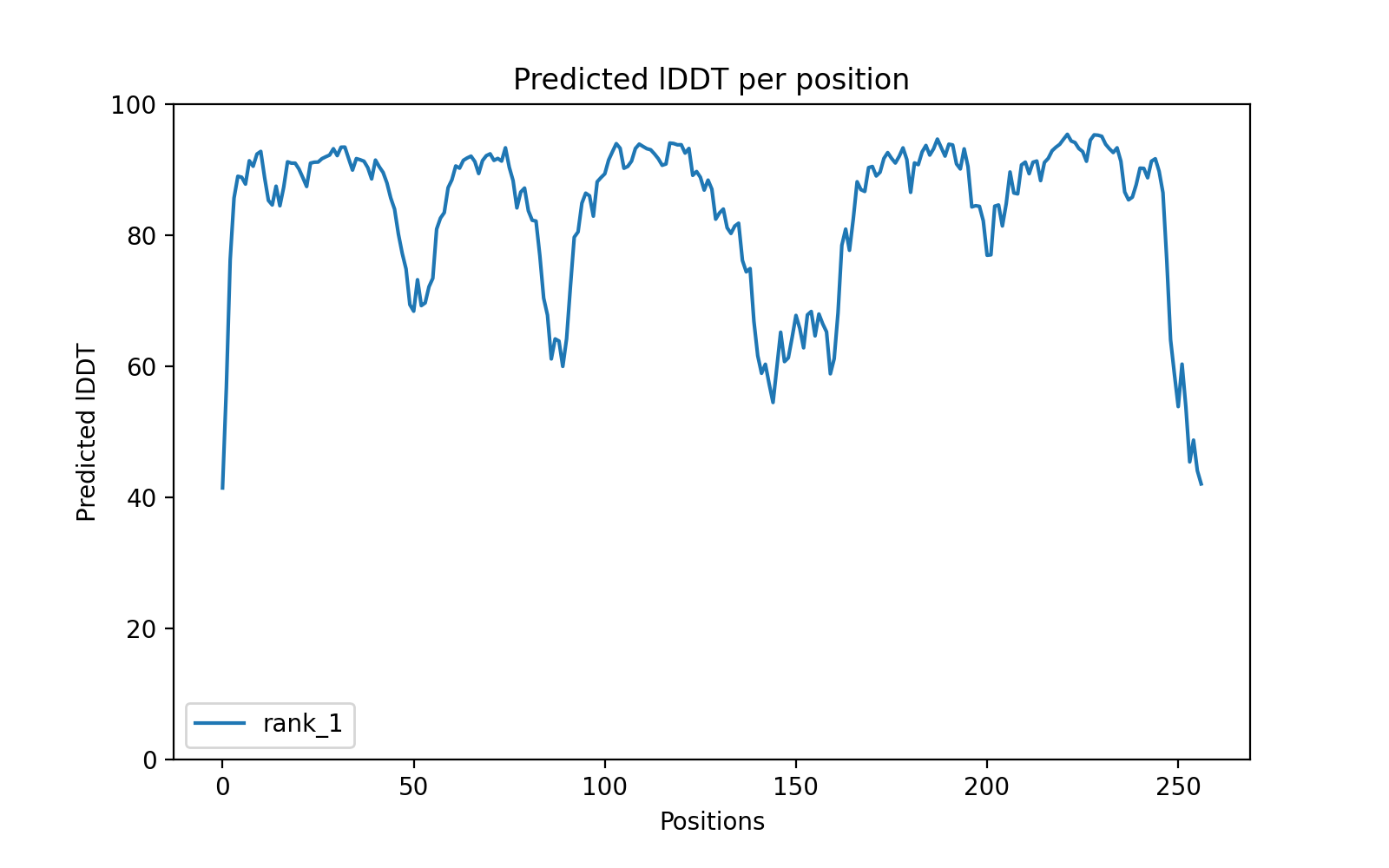

Supplement: S1 File — This file contains the experimentally tested sequences in a zip format. It includes Fasta files, AlphaFold Prediction with PDB file, pLDDT, and more. (ZIP) [file pcbi.1011621.s001.zip › plddt/dg_18_plddt.png]

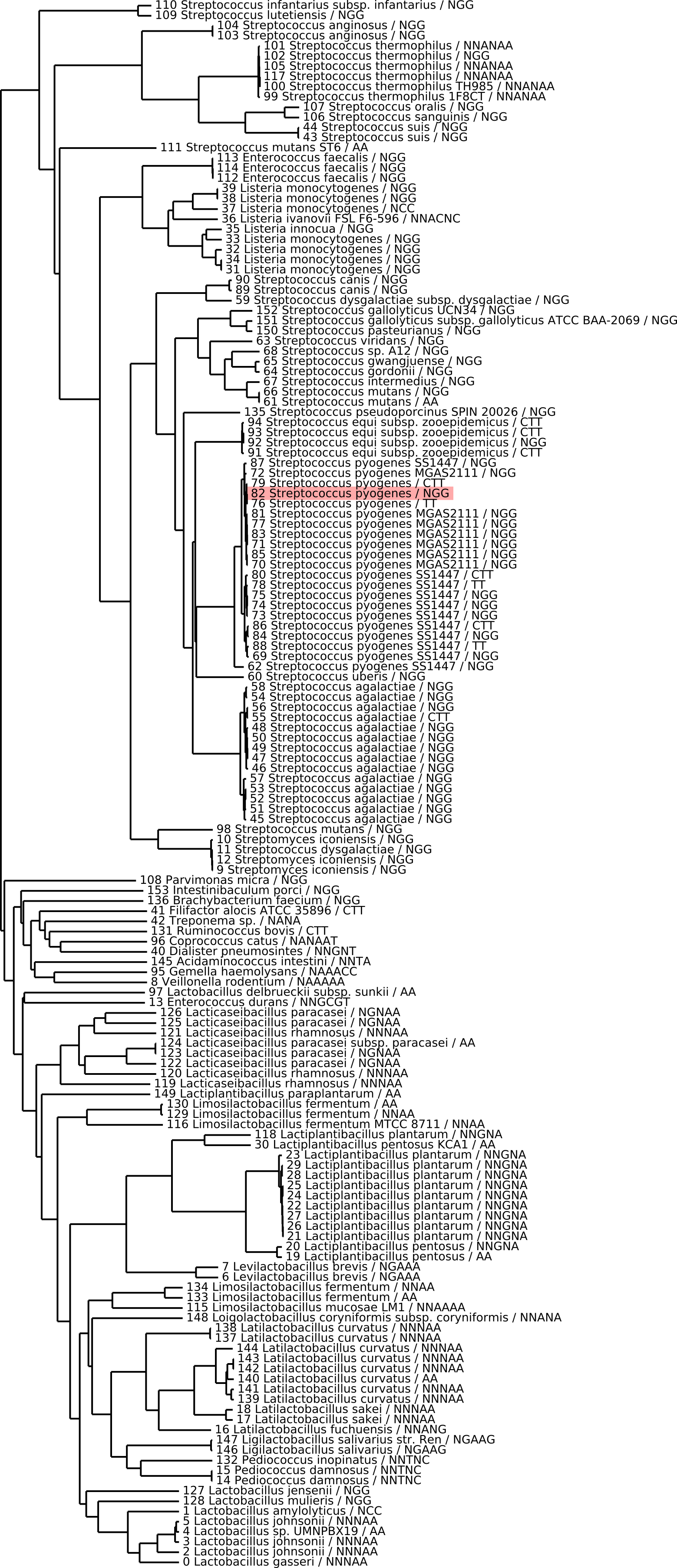

Supplement: S5 File — This PNG file contains the phylogenetic tree of all variants selected from the study by Vink et al. [41]. (TIF) [file pcbi.1011621.s005.tif]
