# Supplementary material for: Computational design of novel Cas9 PAM-interacting domains using evolution-based modelling and structural quality assessment
Source: PLoS Comput Biol. 2023 Nov 17;19(11):e1011621. doi: 10.1371/journal.pcbi.1011621 (PMC10729993; doi:10.1371/journal.pcbi.1011621)
Supplement: S3 File — This PDF file contains additional plots that assess the computational score in combination to predict the activity of variants. (PDF) [file pcbi.1011621.s003.pdf]

S3 file : Additional plots on assessments of computational score

Cyril Malbranke [1,2*], William Rostain [2], Florence Depardieu [2], Simona Cocco [1], Rémi Monasson[1], David Bikard [2]

1 Laboratory of Physics of the Ecole Normale Superieure, PSL Research, CNRS UMR 8023, Sorbonne Université, Université de Paris, Paris, France
2 Institut Pasteur, Université Paris Cité, CNRS UMR 6047, Synthetic Biology, 75015 Paris, France

* cyril.malbranke@phys.ens.fr

1 Prediction of activity using different scores

We performed logistic regressions using different combinations of scores, to assess the interest of combining them linearly. Our results illustrate two things:

- We first used the first batch as a training set and the second batch as a testing set. From this, we show that sequences generated in batch 2 were largely predictable to perform the way they did from the observation of batch 1 (in terms of functionality), as we can see from Fig A **a**. also performed a completely randomized 2-fold cross validation (reproduced 100 times). Predictors trained by performing random cross validation show that adding features to the RBM energy such as the FoldX score, AlphaFold score and the PAM classifier tend to improve the classification of functional sequences. In particular, the AUROC score reached 0.908 for the combination of the RBM, FoldX and AlphaFold2 scores.

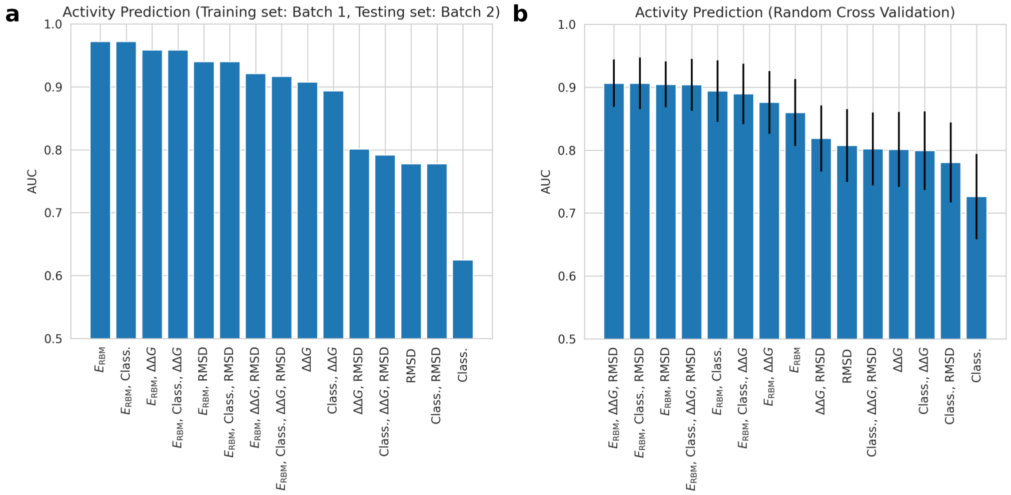

Fig A. a: Classification of variant activity $(\mathrm{n.r} \geq 0.5)$ using logistic regression. The training set is the first batch, the testing set is the second batch. **b**: Activity prediction $(\mathrm{mcherry} \geq 0.5)$ trained and tested through cross validation (completely random, 2 fold, reproduced 100 times).

2 Prediction of activity through different scores

In the main paper, we chose to display the more conclusive scores: E_{RBM}, classifier score, and FoldX $\Delta\Delta\mathrm{G}$. Here, we provide a complete list of scores evaluated in this work:

- E_{RBM}: The RBM energy, with

$$P_{\mathrm{RBM}}(x) = \frac{e^{-E_{\mathrm{RBM}}(x)}}{Z}$$

  bein gthe probability that the variant belongs to the distribution of protein sequences in the family with the trained RBM model.

- Classifier: The score given by the classifier trained along the RBM to the recognition of the TGG motif by the protein sequence.

- AlphaFold2 RMSD: computed using the TM-score software [**?**], this measures the average distance between the backbone atoms of the two protein structures, providing an assessment of how similar or dissimilar two models are. In our case, the AlphaFold2 generated model of the wild-type and that of the variant.

- AlphaFold2 pLDDT: The mean pLDDT is the average of the pLDDT scores across all amino acid residues in a predicted protein structure, providing an overall measure of the model's confidence in the accuracy of the entire structure.

- FoldX $\Delta\Delta\mathrm{G}$ is a value that represents the change in free energy between WT and the variant. It indicates how the mutation we performed affected stability

- FoldX interaction energy: the value that quantifies the affinity between a protein and a ligand, in our case between the PAM-interacting domain and the DNA strands.

In Fig B**a** we show the sequences ordered by each of the metrics. We see that the interaction energy and the pLDDT performed overall more poorly than the other metrics that we proposed in the main paper.

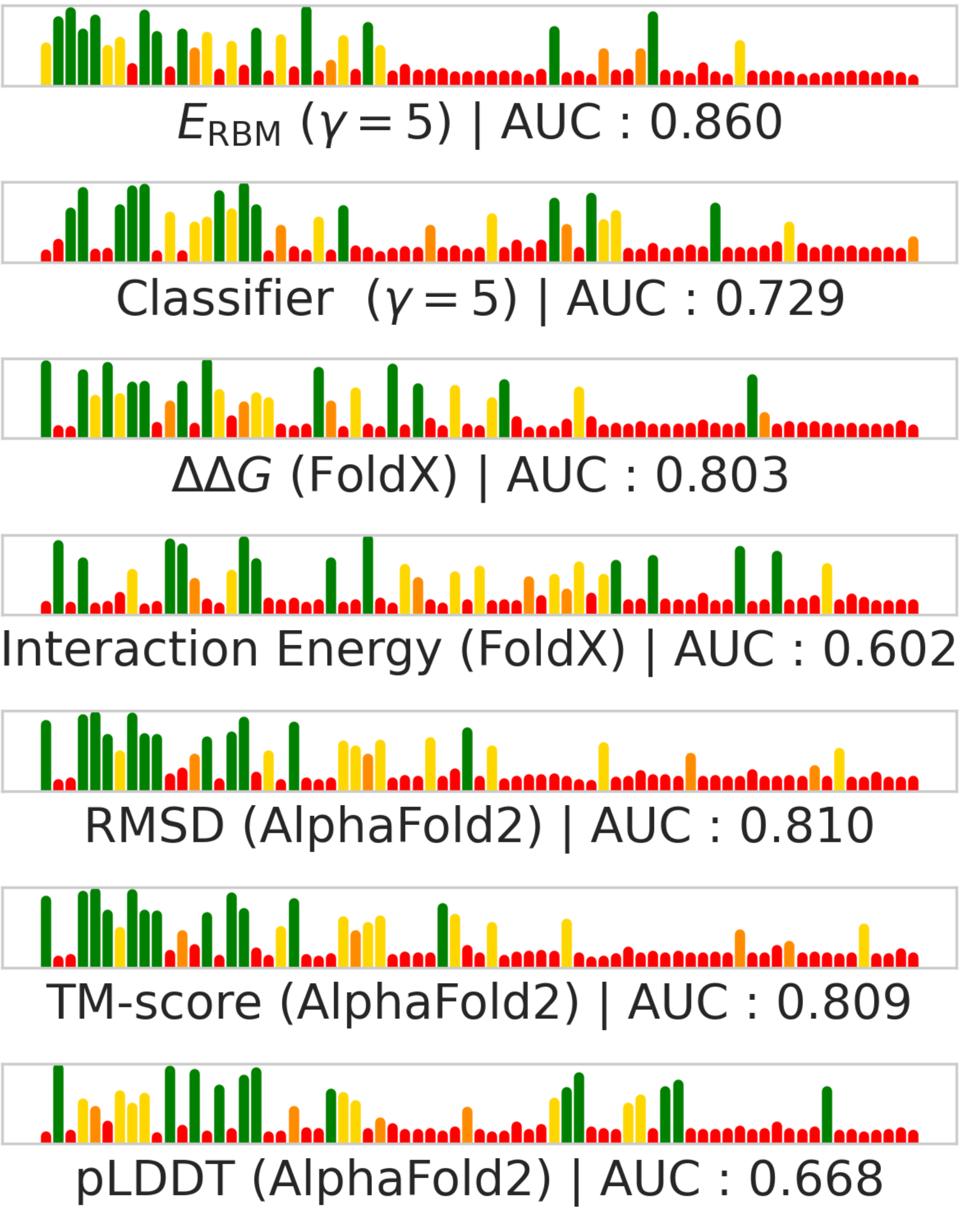

Fig B. We reproduce here some of the scores we selected in that paper as well as other scores we considered but performed less well than the other ones (interaction energy, TM-score, pLDDT)

3 Mutations on critical sites

We assessed whether our method was able to perform mutations on critical sites or whether it was focusing on positions with little importance for protein function or stability. To do so we identified two types of "critical" residues :

- **Residues at the binding interface with DNA**: we used the PDB structure to identify residues within 5 Å of any DNA atom. 18 such residues were identified. We then compared the mutation rate of these sites to the mutation rate on other sites, and we noticed (Fig C) that: (i) these sites were mutated at the same rate as other sites, (ii)

mutations at these sites were not more likely to degrade activity than mutations at other sites. The Spearman correlation between the share of mutations at the DNA interface and the activity is 0.22 (p-value of 0.05) and is lower than the correlation between the share of total residues mutated and the activity ($\rho = 0.36$)

- **Residues critical for protein stability**: We used FoldX's AlaScan to determine which sites were critical for protein stability. This method mutates every single residue to Alanine and computes changes in free energy to evaluate how the stability of the protein depends on each residue . The positions that correspond to destabilizing mutations ($\Delta\Delta G > 0.8\text{kJ.mol}^{-1}$) are the residues critical for protein stability. We identified 113 such residues . In Fig D, we notice that stabilizing residues are slightly less mutated on average than the rest of the protein. Overall, mutations on critical residues do not seem to degrade enzyme activity more than mutations at other positions. The correlation between the share of mutations at critical sites and the activity ($\rho = 0.32$) is similar to the correlation between the share of total residues mutated and the activity ($\rho = 0.36$)

These two observations then tend to show that the method developed seems to perform well in mutating critical sites both at the interface with DNA and important for protein stability.

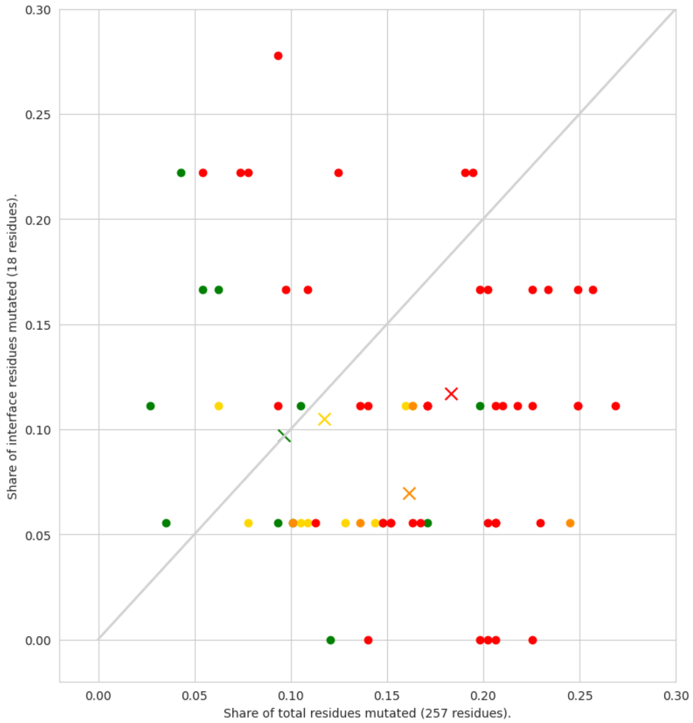

Fig C. The share of mutated residues at the interface with DNA (over the 18 residues) are displayed as a function of the share of total residues mutated (over the 257 residues). From red to green are the four levels of activity of the variants. No strong correlation can be seen between the mutation at the interface and the activity of the variants. We also computed the mean over each level of activity of the share of residues mutated over the full sequence and over the residues on the interface (crosses from red to green). As we can see, the functional variants have on average the same share of mutations **on interface** than the non-functional one.

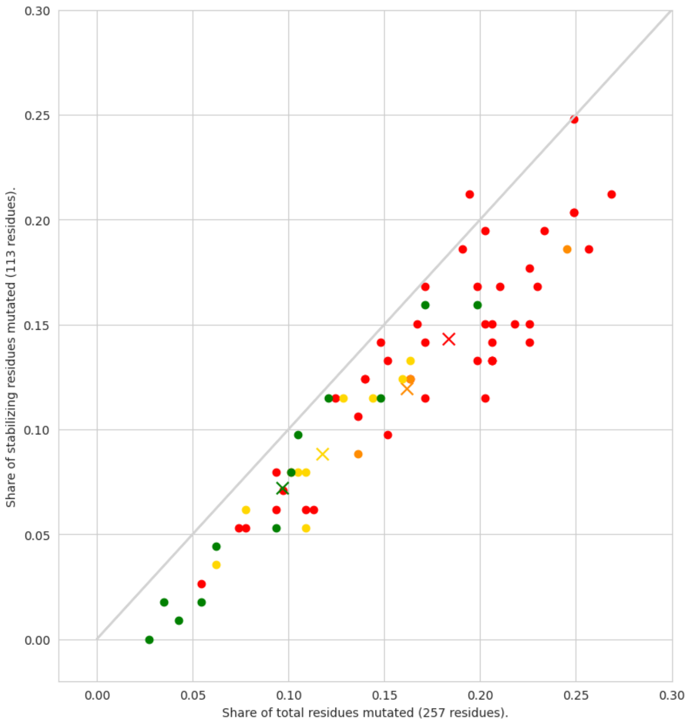

Fig D. The share of mutated residues important for protein stability (over the 113 residues) are displayed as a function of the share of total residues mutated (over the 257 residues). From red to green are the four levels of activity of the variants. We can see that the residues important for protein stability are slightly less mutated (the same share of mutation would correspond to being on the gray line). However, if the share of mutations on stabilizing residues affects the activity of the variant, it does not seem that this has more effect than the overall share of mutations on the full sequence.

.
