# Supplementary material for: Computational design of novel Cas9 PAM-interacting domains using evolution-based modelling and structural quality assessment
Source: PLoS Comput Biol. 2023 Nov 17;19(11):e1011621. doi: 10.1371/journal.pcbi.1011621 (PMC10729993; doi:10.1371/journal.pcbi.1011621)
Supplement: Attachment — Submitted filename: PCOMPBIOL-D-23-00836_review.pdf [file pcbi.1011621.s006.pdf]

Comments to the author:

The authors present a work on an approach to protein design where functional data and evolutionary information are utilized in a Restricted Boltzmann Machine. The objective is to learn a sequence model of a protein family. Additionally, the authors employ physics-grounded modeling to investigate variants generated by the model and experimentally test some of them. This study is interesting because it demonstrates how a simple model can integrate information from diverse sources to generate a specialized computational machine. Thus, it serves as an example of the synergy between explainable machine learning and physics-grounded modeling.

However, this work is let down by its presentation. Several passages are left unexplained, and some contain errors that can be rectified. In general reading the manuscript and checking the supplementary materials it becomes clear that the presentation does not do justice to the work carried out. I suggest that the authors carefully revise the manuscript when implementing the suggested changes. Alternatively, being aware that the ones knowing better the presented work are the authors themselves, authors can provide extensive deep and clear explanations as to why they are not applying the changes. Finally, beyond the changes, I suggest authors meticulously check the coherence of the text and that every piece of information is referenced or shortly explained.

It is important to note that all the criticisms mentioned above are solely based on how this work was presented. To reiterate, I believe the tool itself represents an important development. Therefore, addressing at least partially the following concerns and improving the clarity of the paper would greatly benefit its overall impact.

Major comments:

"Our approach enabled the generation of functional variants of the Cas9 PID that differ by more than 50 amino-acids from natural sequences"
→ Having 50 mutations on a protein does not imply high engineering power for a method. It is possible that those residues were exposed to the solvent and mutated toward similar amino acids, which does not provide substantial insight into the power of your method. Mutations in crucial regions for stability or DNA interaction hold more significance. It would be more interesting to know the percentage of functional variants generated by your method that had mutations at the DNA interface and the percentage that had mutations at positions relevant for the stability of the wild-type (WT). You can determine the former by measuring the distance between the residue and DNA, and the latter by employing tools like FoldX and AlaScan on the WT. This is more informative regarding the efficacy of your method than simply knowing that 50 residues have changed.

"We also used AlphaFold2 to assess the quality of the proteins generated with our method."
→ Alphafold2 (AF2) can hardly be seen as a tool to assess the quality of the proteins generated with your method. AF2 is a computational tool to get accurate models given a protein sequence. It is not a protein design tool, and it is not yet clear to what degree it can be used for this purpose. It is difficult to explain

how AF2 can be used to assess the quality of your design, especially when your molecule's function is to bind DNA, which AF2 cannot model. As far as I understand from your work, you modeled variants with AF2 (without modeling the DNA because this is not possible), and you "compared" (I had to guess, by measuring RMSD) the WT model with your variants. This approach has so many implications that make it impossible to consider that AF2 can be used here to assess the quality of the models you generated. It makes sense that you use AF2 to generate models (which is what AF2 is incredibly good at) to compare with the ones you use in your pipeline and determine their agreement, but I would not say you used it to assess the quality of your proteins. You should explain why you chose to use RMSD with WT instead of the real AF2 score (the plDDT that is generated both for the structure and for the residues) or implement that instead.

"To do so, we used Constrained Langevin Dynamics to generate 120 protein sequences per RBM, with an RBM energy comparable to the one of the reference sequence (SpyCas9) and numbers of mutations ranging from 10 to 50."
→ as mentioned before, here is a good place to give some more information on the quality of the mutations your approach generated (how many are in contact with DNA? How many at the surface? etc..)

"The evaluation was performed by taking the structure of the Cas9-PID in complex with DNA (PDB-ID : 5y36). We then replaced the amino acids by their values in the generated sequences, and repaired the structure by fixing the side chains. Functional sequences are expected to have a resulting FoldX energy close to that of the WT sequence."
→ The procedure for utilizing FoldX correctly is as follows. Firstly, download the 5y36 structure and apply RepairPDB to fix any structural issues. Next, design the mutations corresponding to the ones generated by your algorithm on the repaired structure using BuildModel. Write all the mutations of each variant in the same individual_list.txt file so you'll have one mutant per sequence. After executing BuildModel, a *Diff*****.fxout* file will be generated, providing the most accurate DDG (free energy change) of stability for your variant compared to the wildtype (5y36). For stability analysis, DDGs greater than +0.8 kcal/mol are considered destabilizing, while DDGs less than -0.8 kcal/mol are considered stabilizing.
If you wish to assess the impact of mutations on DNA interaction, you can utilize FoldX AnalyseComplex (available at https://foldxsuite.crg.eu/command/AnalyseComplex). In this case, you should run the command on two PDB files: the mutated one and its corresponding FoldX wildtype (the PDB file produced by BuildModel, whose name starts with "WT"), the difference in Interaction energy can be calculated using the two generated *Summary****fxout* files (one for the wt one for the mutant).
It is important to note that selecting mutants based on FoldX energy and subsequently repairing structures after mutation is a significant mistake, as it diminishes the comparability of variant structures flattering the dependence of the predicted energy on the different residue identities. Make sure to explain how you compute DDGs in the methods section.
All FoldX DDGs should be obtained as explained above.

Fig.9

→The caption of this figure requires a complete rewrite. There are multiple spelling errors, and the usage of "AlphaFold RMSD" is unclear since it has not been defined previously (it just appears 2 more times in the text and there is no reference to methods or supplementary). I inferred that it might refer to the "*comparison*" between WT and mutant models mentioned earlier in the text. However, I still recommend substituting this value with the AF2 plDDT score if it makes sense, which is an actual metric provided by the tool. If you strongly believe that this RMSD is a better estimator of the effect of mutations on your target protein, it is essential to define what it is beforehand, either in the methods section or elsewhere.

Furthermore, I suggest incorporating FoldX into this analysis. By using the BuildModel command, you can isolate the effect of each mutation and obtain the relative DDG (free energy change) as the output. This will allow you to assess the impact of individual mutations. Additionally, you can separate the effects on stability from those on DNA interaction, if desired.

Minor comments:

"studying protein interactions with ligands such as other proteins, RNA, DNA, and metals."
→ cite any method (suggestion: https://pubmed.ncbi.nlm.nih.gov/29608705/; https://pubmed.ncbi.nlm.nih.gov/31732673/ ;)

"Numerous efforts have therefore been dedicated over the last decade to both characterize natural variants of Cas9 with other recognition motifs (see Collias et Beisel 2021 [32] for an overview), and engineer Cas9 variants with expanded recognition capabilities [33–35]."
→ Isn't this in conflict with the following sentence in your work: "our evolution-based method is not ideal for generating close variants with modified PAM specificity"? Please clarify or resolve the issue, currently, it seems like you only offer modeling of variants to bind the same target, which is still valuable but this sentence in the introduction made me think you were addressing the same issue.

"As we see, we reach a maximum for intermediate value of γ that outperforms standard RBM ($\gamma = 0$)"
→ state which γ value and explain better why it is significant.

"Our model also outperformed a classifier trained uniquely on the labeled sequence data (high values of γ)"
→ where can I see this? Reference it in the text.

Fig4.a
→ the caption of the figure should explain the figure. I couldn't understand it. What are the 2 red trapeze for? Also if possible would be nice to improve the aesthetic a bit.

"we show two examples of random walks with 5 different chains. We also display in supplementary (S5 file) some additional results on MNIST"

→ reference and provide an explanation of MNIST, and explain why it was useful to apply your approach to this specific dataset. The sentence in its current form lacks clarity and may lead to confusion.

"We then replaced the amino acids by their values in the generated sequences"
→ instead of values I would say 'residue identities'.

"This results indicate that semi-supervised training on functional data, here the PAM recognition sequence, can improve the overall ability of the model to generate proteins that are likely to fold properly."
→ these results indicate that this method produces sequences that are predicted to be stable variants of your protein according to FoldX. But to state this you have to repeat the analysis in the correct way explained above. Also you can also (optionally) show that your method provides variants with affinity to the DNA using AnalyseComplex as explained above.

"These sequences were generated utilizing the representation space of a self-supervised learning Restricted Boltzmann Machine (SSL-RBM) "
→ semi or self supervised?

"We selected 71 sequences spanning different predicted scores to be assessed experimentally (29 with method 1 and 42 with method 2)."
→ you selected those 71 based on what? Even if it was random, explain it.

Fig.5a,b
→ add axis names

"AlphaFold2 is a framework that uses modern neural networks to predict a distance map from a sequence and an alignment of the sequence homologs. This distance map is then used to build a 3-dimensional model of the protein structure through physically motivated methods."
→ I think this sentence is not correct: what do you mean with 'through physically motivated methods'? Just reference the method, it is way better. Also in the text you use AlphaFold and AlphaFold2, which one do you use? Make it uniform in the text.

"On Fig 8a, we display the correlation with the structural"
→ you meant 8b?

"Interestingly, models seemed to capture complementary information, as can be seen in the failure of AlphaFold to spot inactive samples which are correctly predicted as active by the RBM and vice-versa."
→ how can I visualize this observation?

"Second, this motif is recognized by our reference enzyme and by the large majority of close natural variants present in our dataset, as indicated by the comprehensive phylogenetic tree provided in

supplementary (S6 file). Due to these limitations, our evolution-based method is not ideal for generating close variants with modified PAM specificity."
⟶ rephrase to make it more clear

"One promising strategy could involve employing Deep Learning frameworks like ProteinMPNN [60], or other platforms specifically designed to model binding interactions [61]. These frameworks could serve as viable alternatives to FoldX, offering enhanced flexibility and potentially more robust modeling capabilities. "
⟶Proteinmpnn is a sequence generator for input structures, while FoldX is a force field that calculates DDG (free energy change) for structure models. If proteinmpnn generates sequence variants for a given structure, its function seems to be more similar to the RBM (Restricted Boltzmann Machine) presented in this manuscript rather than FoldX. Also could you clarify what you mean by "more flexibility and robust modeling"? It would be beneficial to engage in a more in-depth discussion on future aspects or, if not possible, omit this part of discussion altogether.

Supp mat
⟶ provide detailed explanation for all tables, what are the columns?
