# Supplementary material for: Computational design of novel Cas9 PAM-interacting domains using evolution-based modelling and structural quality assessment
Source: PLoS Comput Biol. 2023 Nov 17;19(11):e1011621. doi: 10.1371/journal.pcbi.1011621 (PMC10729993; doi:10.1371/journal.pcbi.1011621)
Supplement: Attachment — Submitted filename: answer_to_reviewers.pdf [file pcbi.1011621.s007.pdf]

We wish to thank the reviewers for the very useful comments they provided. Furthermore, we hope this improved version meet well the comments of the reviewers.

Green: comment from reviewer
Blue: answer
Gray: reproduction of important changes in the manuscript

We also provide a diff file between old and new main attached as a separate pdf.

Reviewer #1: Malbranke et al. present a framework for integrating sequence information, structural modeling scores and experimental data into protein design. The authors use a Restricted Boltzmann Machine (RBM) to learn a continuous representation space for a protein family, in this particular study the Cas9 PAM-interacting domain (PID). For incorporating functional data, a semi-supervised learning (SSL) RBM was trained, aiming to improve the statistical representation of the protein family learned by the RBM. For designing new sequences of the Cas9 PID, the latent space learned by the RBM is explored using Langevin dynamics guided by constraints such as RBM score and FoldX force field energy. 71 designed variants were experimentally tested; 21 were functional and 6 had an improved activity compared to the wildtype protein. Some designs that retained function had up to 50 sequence changes compared to wildtype. Interestingly, sequences designed with both constraints, RBM score and FoldX energy, showed the highest activity on average.

The paper is well written and the results and methods are well explained. The study nicely demonstrates the combination of protein sequence machine learning models with structure-based modeling for protein design.
I have only a few minor comments / questions.

Minor comments:
Can the authors comment on how the number of neurons in the hidden layer (200 Gaussian units) was chosen?

Thank you for the question, we added a small note on that in methods:

“The number of neurons in the hidden layer (200) was fixed arbitrarily at an early stage by a quick exploration of the number of Gaussian units. We built a training/validation set on PF16595, using MMSEQS2 to ensure that sequences in the validation set were different enough from sequences in the training set, and we optimized the number of hidden units so to avoid overfitting (when the number of hidden units was too high).”

Can the authors clarify which FoldX score ($\Delta\Delta G$?) and which AlphaFold score (pLDDT or pTM ?) were used for sequence function prediction and/or comparison with the activity of tested variants?

Thanks to the comments of reviewer 2 we realized that there was a better way to compute a FoldX score than the one we used initially. We have now followed the detailed advice of reviewer 2 to compute the $\Delta\Delta G$ and have updated the text and figures accordingly (see more details in our reply to reviewer 2).

Regarding the AlphaFold scores, we used the RMSD (root-mean-squared deviation) between the predicted structure of the variant and the predicted structure of the WT. The pLDDT score was also tested, but was a poorer predictor of Cas9 activity than the RMSD score. We now provide additional details in the manuscript:

“We further attempted to compare the information obtained by FoldX with the more recent protein structure prediction tool AlphaFold2 \cite{jumper2021a}. For each experimentally tested variant, we predicted a model of the structure (using ColabFold \cite{mirdita2022}). This model was then aligned with the AlphaFold2-predicted model for the wild-type protein using the TMscore program \cite{zhang2004}. From this alignment, we derived the root-mean-square deviation (RMSD) between the models. RMSD measures the root of the mean squared distance between the backbone atoms of the two protein models. We also collected from AlphaFold2 the average pLDDT score for each model, pLDDT is a per-residue confidence score indicating the reliability of the predicted position for each amino acid in the protein structure, which in the past has been shown to correlate well with variant activity. This score can be found in \nameref{file:s3}. We found that the RMSD (AUC = 0.810) performed better than the pLDDT (AUC = 0.668) to predict the activity of experimentally measured variants.”

Page 3, line 1: “PID from SpCas9” should be “PID from SpyCas9”

We uniformized the notations so that the SpyCas9 notation is the only one used in the paper

Page 4, Fig 1 caption: “gene contruct” should be “gene construct”
Page 4, Fig1 caption: “exchance” should be “exchange”
Page 11, line 8: “bottom-right part” should be “upper-left part” I think.

Thank you very much for these remarks, we corrected these mistakes in the new manuscript

Reviewer #2

The authors present a work on an approach to protein design where functional data and evolutionary information are utilized in a Restricted Boltzmann Machine. The objective is to learn a sequence model of a protein family. Additionally, the authors employ physics-grounded modeling to investigate variants generated by the model and experimentally test some of them. This study is interesting because it demonstrates how a simple model can integrate information from diverse sources to generate a specialized computational machine. Thus, it serves as an example of the synergy between explainable machine learning and physics-grounded modeling.

However, this work is let down by its presentation. Several passages are left unexplained, and some contain errors that can be rectified. In general, reading the manuscript and checking the supplementary materials, it becomes clear that the presentation does not do justice to the work carried out. I suggest that the authors carefully revise the manuscript when implementing the suggested changes.

Alternatively, being aware that the ones knowing better the presented work are the authors themselves, authors can provide extensive deep and clear explanations as to why they are not applying the changes. Finally, beyond the changes, I suggest authors meticulously check the coherence of the text and that every piece of information is referenced or shortly explained.
It is important to note that all the criticisms mentioned above are solely based on how this work was presented. To reiterate, I believe the tool itself represents an important development. Therefore, addressing at least partially the following concerns and improving the clarity of the paper would greatly benefit its overall impact.

Major comments:

- “Our approach enabled the generation of functional variants of the Cas9 PID that differ by more than 50 amino-acids from natural sequences”

→ Having 50 mutations on a protein does not imply high engineering power for a method. It is possible that those residues were exposed to the solvent and mutated toward similar amino acids, which does not provide substantial insight into the power of your method. Mutations in crucial regions for stability or DNA interaction hold more significance. It would be more interesting to know the percentage of functional variants generated by your method that had mutations at the DNA interface and the percentage that had mutations at positions relevant for the stability of the wild-type (WT). You can determine the former by measuring the distance between the residue and DNA, and the latter by employing tools like FoldX and AlaScan on the WT. This is more informative regarding the efficacy of your method than simply knowing that 50 residues have changed.

See reply below

- “To do so, we used Constrained Langevin Dynamics to generate 120 protein sequences per RBM, with an RBM energy comparable to one of the reference sequence (SpyCas9) and numbers of mutations ranging from 10 to 50.”
→ as mentioned before, here is a good place to give some more information on the quality of the mutations your approach generated (how many are in contact with DNA? How many at the surface?

Thank you for these two important comments. Following the advice of the reviewer we used FoldX to identify residues at the DNA interface and residues decisive for protein stability that were mutated in our experimentally measured variants. We identified 18 residues at the interface and more than 113 residues critical for protein stability. The two plots reproduced below were added to the supplementary materials and discussed there:

“We assessed whether our method was able to perform mutations on critical sites or whether it was focusing on positions with little importance for protein function or stability. To do so we identified two types of "critical" residues :
\begin{itemize}

\item \textbf{Residues at the binding interface with DNA}: we used the PDB structure to identify residues within 5 \angstrom~of any DNA atom. 18 such residues were identified. We then compared the mutation rate of these sites to the mutation rate on other sites, and we noticed (Fig~\ref{fig:supp_interfacevstotal}) that: (i) these sites were mutated at the same rate as other sites, (ii) mutations at these sites were not more likely to degrade activity than mutations at other sites. The Spearman correlation between the share of mutations at the DNA interface and the activity is 0.22 (p-value of 0.05) and is lower than the correlation between the share of total residues mutated and the activity ($\rho = 0.36$)
\item \textbf{Residues critical for protein stability}: We used FoldX's AlaScan to determine which sites were critical for protein stability. This method mutates every single residue to Alanine and computes changes in free energy to evaluate how the stability of the protein depends on each residue . The positions that correspond to destabilizing mutations ($\Delta\Delta G > 0.8 \mathrm{kJ.mol^{-1}}$) are the residues critical for protein stability. We identified 113 such residues . In Fig~\ref{fig:supp_stabilizingvstotal}, we notice that stabilizing residues are slightly less mutated on average than the rest of the protein. Overall, mutations on critical residues do not seem to degrade enzyme activity more than mutations at other positions. The correlation between the share of mutations at critical sites and the activity ($\rho = 0.32$) is similar to the correlation between the share of total residues mutated and the activity ($\rho = 0.36$)
\end{itemize}
These two observations then tend to show that the method developed seems to perform well in mutating critical sites both at the interface with DNA and important for protein stability."

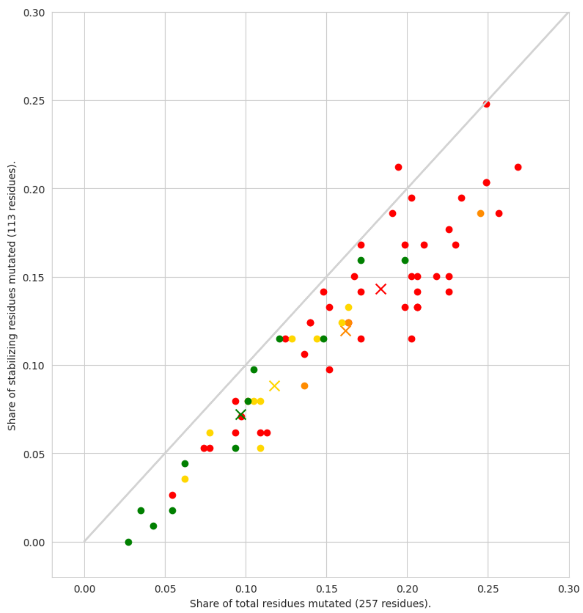

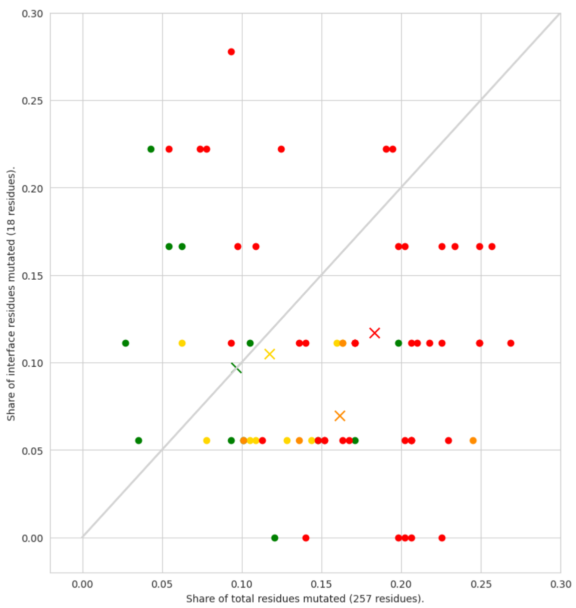

and discussed in the main text as follow:

"Finally, we can also stress that our method produced functional variants with mutations at the DNA binding interface, as well as mutations at positions identified as critical for protein

stability, with these mutations being only slightly less frequent than mutations at other positions (details can be found in supplementary (\nameref{file:s3})). This indicates that the method can perform mutation on critical area of the protein while retaining the activity of the variants."

- "We also used AlphaFold2 to assess the quality of the proteins generated with our method."

→ Alphafold2 (AF2) can hardly be seen as a tool to assess the quality of the proteins generated with your method. AF2 is a computational tool to get accurate models given a protein sequence. It is not a protein design tool, and it is not yet clear to what degree it can be used for this purpose. It is difficult to explain how AF2 can be used to assess the quality of your design, especially when your molecule's function is to bind DNA, which AF2 cannot model. As far as I understand from your work, you modeled variants with AF2 (without modeling the DNA because this is not possible), and you "compared" (I had to guess, by measuring RMSD) the WT model with your variants. This approach has so many implications that make it impossible to consider that AF2 can be used here to assess the quality of the models you generated. It makes sense that you use AF2 to generate models (which is what AF2 is incredibly good at) to compare with the ones you use in your pipeline and determine their agreement, but I would not say you used it to assess the quality of your proteins. You should explain why you chose to use RMSD with WT instead of the real AF2 score (the plDDT that is generated both for the structure and for the residues) or implement that instead.

See reply below

→The caption of this figure requires a complete rewrite. There are multiple spelling errors, and the usage of "AlphaFold RMSD" is unclear since it has not been defined previously (it just appears 2 more times in the text and there is no reference to methods or supplementary). I inferred that it might refer to the "comparison" between WT and mutant models mentioned earlier in the text. However, I still recommend substituting this value with the AF2 plDDT score if it makes sense, which is an actual metric provided by the tool. If you strongly believe that this RMSD is a better estimator of the effect of mutations on your target protein, it is essential to define what it is beforehand, either in the methods section or elsewhere.

Thank you for this helpful comment, and apologies for the lack of clarity in defining our scoring metrics. Indeed, as you properly understood, we used AlphaFold2 to generate one model for the WT and one model for each of the variants generated.

For each:

- we collected the pLDDT and used it as a score for assessing the quality of our variants
- we used structural alignments to align the two models together and compute the RMSD (root mean squared deviation between corresponding atoms of the model).

We were at first agnostics regarding which score would perform best. We noticed that the RMSD allowed us to predict the activity of our generated variants better than the pLDDT score, and therefore only showed the RMSD in the main text.

Here are the steps we took to clarify these points in the manuscript:

-> We better define RMSD in the paper.

“We further attempted to compare the information obtained by FoldX with the more recent protein structure prediction tool AlphaFold2 \cite{jumper2021a}. For each experimentally tested variant, we predicted a model of the structure (using ColabFold \cite{mirdita2022}). This model was then aligned with the AlphaFold2-predicted model for the wild-type protein using the TMscore program \cite{zhang2004}. From this alignment, we derived the root-mean-square deviation (RMSD) between the models. RMSD measures the root of the mean squared distance between the backbone atoms of the two protein models. We also collected from AlphaFold2 the average pLDDT score for each model, pLDDT is a per-residue confidence score indicating the reliability of the predicted position for each amino acid in the protein structure, which in the past has been shown to correlate well with variant activity. This score can be found in \nameref{file:s3}. We found that the RMSD (AUC = 0.810) performed better than the pLDDT (AUC = 0.668) to predict the activity of experimentally measured variants.”

-> We rewrote the caption

“Each panel represents the precision in detecting inactive variants using different scores: RBM energy (\textbf{a}), AlphaFold2 RMSD (\textbf{b}), and FoldX's $\Delta\Delta G$ (\textbf{c}). These metrics are plotted against the mutation's location within the protein sequence and structure. DNA regions are highlighted in blue. Green signifies regions where the deleterious impact of mutations is accurately predicted. Conversely, red indicates regions where predictions miss the mark. Areas with insufficient data (less than five variant with a mutation at this position) are marked in gray. Notably, the RBM energy offers precise predictions at the DNA-binding interface, in contrast to AlphaFold2 RMSD and $\Delta\Delta G$, which tend to be less accurate at these sites.”

-> Furthermore, we added a part detailing a bit more some metrics tested in the supplementary.

“In the main paper, we chose to display the more conclusive scores: E_{RBM}, classifier score, and FoldX $\Delta \Delta \mathrm{G}$. Here, we provide a complete list of scores evaluated in this work:
\begin{itemize}
\item E_{RBM}: The RBM energy, with \[
P_{\mathrm{RBM}}(x) = \frac{e^{-E_{\mathrm{RBM}}(x)}}{Z}
\] bein gthe probability that the variant belongs to the distribution of protein sequences in the family with the trained RBM model.
\item Classifier: The score given by the classifier trained along the RBM to the recognition of the TGG motif by the protein sequence.

\item AlphaFold2 RMSD: computed using the TM-score software \cite{zhang2004}, this measures the average distance between the backbone atoms of the two protein structures, providing an assessment of how similar or dissimilar two models are. In our case, the AlphaFold2 generated model of the wild-type and that of the variant.
\item AlphaFold2 pLDDT: The mean pLDDT is the average of the pLDDT scores across all amino acid residues in a predicted protein structure, providing an overall measure of the model's confidence in the accuracy of the entire structure.
\item FoldX $\Delta \Delta \mathrm{G}$ is a value that represents the change in free energy between WT and the variant. It indicates how the mutation we performed affected stability
\item FoldX interaction energy: the value that quantifies the affinity between a protein and a ligand, in our case between the PAM-interacting domain and the DNA strands.
\end{itemize}
In Fig~\ref{fig:supp_all_scores}\textbf{a} we show the sequences ordered by each of the metrics. We see that the interaction energy and the pLDDT performed overall more poorly than the other metrics that we proposed in the main paper."

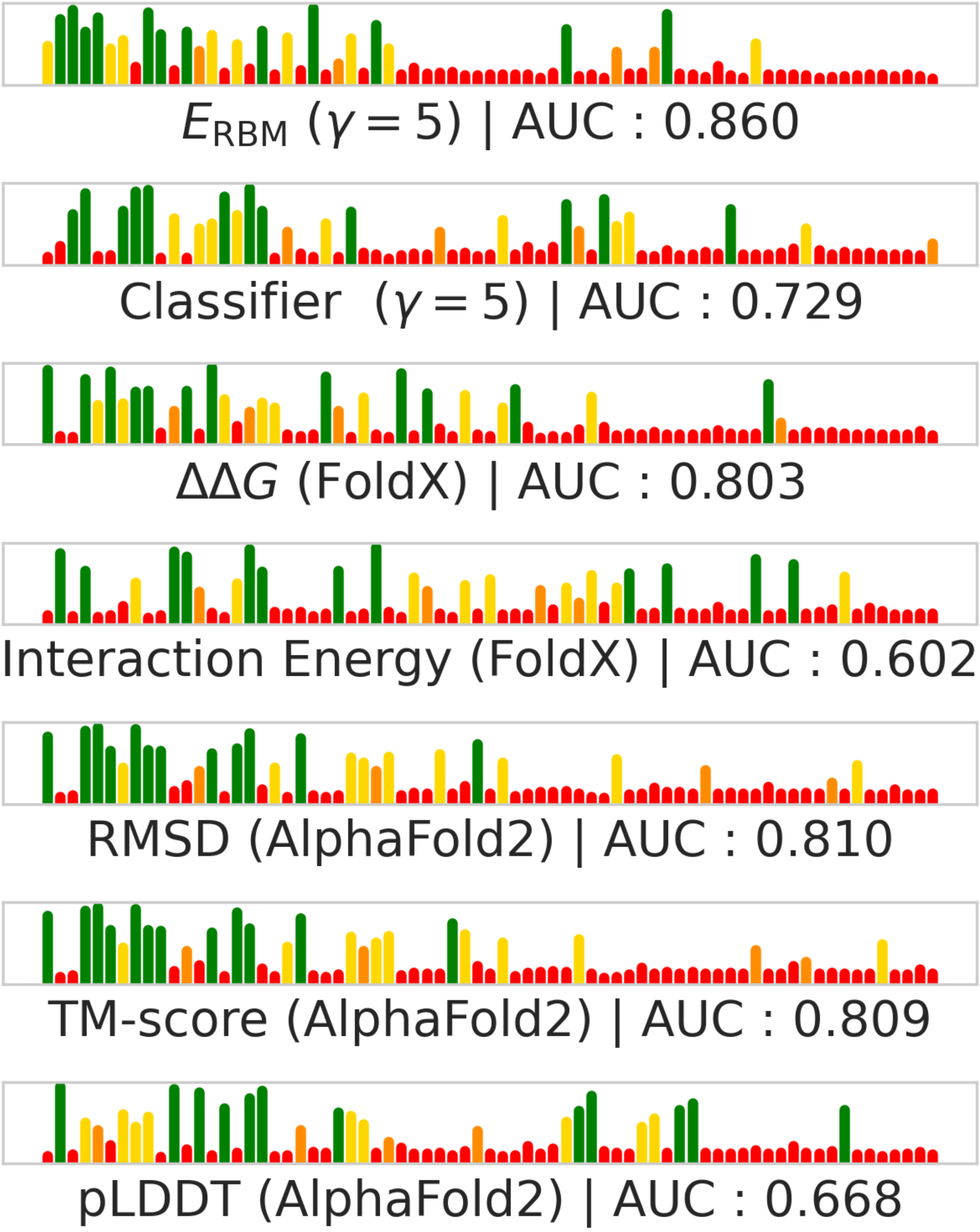

- “The evaluation was performed by taking the structure of the Cas9-PID in complex with DNA (PDB-ID : 5y36). We then replaced the amino acids by their values in the generated sequences, and repaired the structure by fixing the side chains. Functional sequences are expected to have a resulting FoldX energy close to that of the WT sequence.”

→ The procedure for utilizing FoldX correctly is as follows. Firstly, download the 5y36 structure and apply RepairPDB to fix any structural issues. Next, design the mutations corresponding to the ones generated by your algorithm on the repaired structure using BuildModel. Write all the mutations of each variant in the same individual_list.txt file so you’ll

have one mutant per sequence. After executing BuildModel, a Diff.fxout file will be generated, providing the most accurate DDG (free energy change) of stability for your variant compared to the wild type (5y36). For stability analysis, DDGs greater than +0.8 kcal/mol are considered destabilizing, while DDGs less than -0.8 kcal/mol are considered stabilizing you wish to assess the impact of mutations on DNA interaction, you can utilize FoldX AnalyseComplex (available at https://foldxsuite.crg.eu/command/AnalyseComplex). In this case, you should run the command on two PDB files: the mutated one and its corresponding FoldX wild type (the PDB file reduced by BuildModel, whose name starts with "WT"), the difference in Interaction energy can be calculated using the two generated Summary.fxout files (one for the wt one for the mutant).

Make sure to explain how you compute DDGs in the methods section.
All FoldX DDGs should be obtained as explained above.

It is important to note that selecting mutants based on FoldX energy and subsequently repairing structures after mutation is a significant mistake, as it diminishes the comparability of variant structures flattering the dependence of the predicted energy on the different residue identities.

During generation phase on batch #1 we used FoldX to perform mutations without using the BuildModel function to save computational time. For the revision of the paper we followed the reviewer suggestions using BuildModel and AnalyseComplex and we noticed that:

1) The DDG computed was a slightly better predictor of enzyme activity (the AUC improved from 0.77 to 0.8)
2) The Interaction energy was a bad predictor of enzyme activity which might be linked to the fact that the binding site is not directly impacted by most mutations.
   You can see in the supplementary (S3 file) of the manuscript the interaction energy for the tested variants
3) The figures and methods of the manuscript were updated accordingly: we replaced the old energy which was less precisely calculated, and not subtracted by the free energy of the wild type, by the canonical DDG in all figures (Fig 5, 6, 8). We also updated the captions accordingly.

In method, we detailed the methods used for variant generation and for the final evaluation :

“\subsection{Computation of FoldX $\Delta \Delta \mathrm{G}$}

We collected the PDB structure of Cas9 PAM-Interacting domain (PDBid: 5y36) in complex with DNA and used the RepairPDB function from FoldX to optimize the quality of the model.

\subsubsection{Quick computation for first method design}

As explained in section~\ref{sec:expe-protocal}, the first method we propose requires the quick evaluation of the stability of multiple variants with few differences to the variant of the previous step. To achieve this, we mutated the residues and used the Optimize function from FoldX to fix the side-chains. We then used the Stability function to evaluate the stability of the variant.

\subsubsection{Precise computation a posteriori}

All variants that were experimentally tested as well as additional untested variants had their stability re-evaluated using the FoldX framework. For each variant, we build the list of mutations to the wild-type and used the BuildModel function to evaluate stability. You can see these stability estimates on Fig~\ref{fig:43_expe_results1bis} for the experimentally tested variants and on Fig~\ref{fig:ssl-rbm2} for the untested ones.”

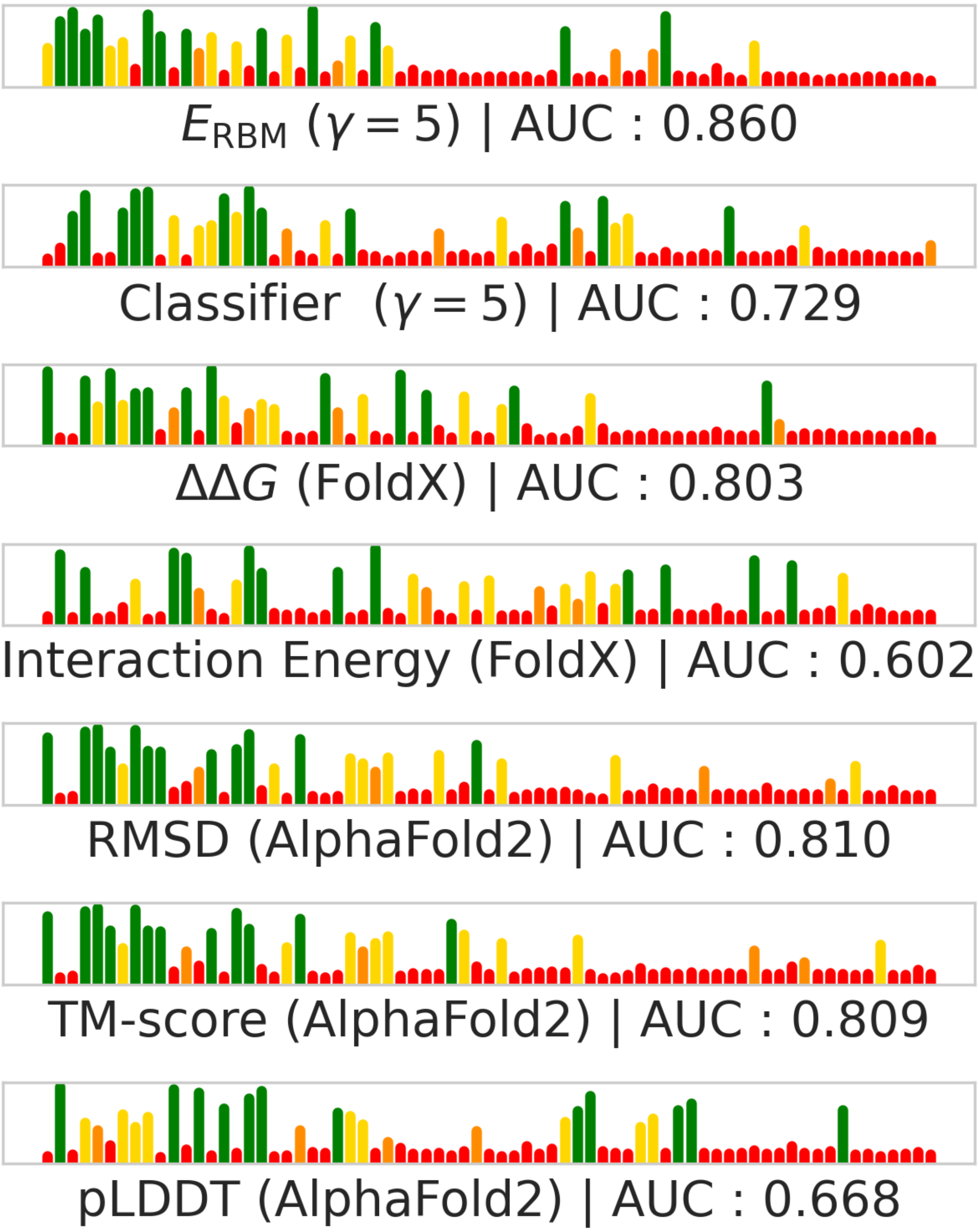

- Fig.9

Furthermore, I suggest incorporating FoldX in- “Numerous efforts have therefore been dedicated over the last decade to both characterize natural variants of Cas9 with other recognition motifs (see Collias et Beisel 2021 [32] for an overview), and engineer Cas9 variants with expanded recognition capabilities [33–35].”
→ Isn't this in conflict with the following sentence in your work: "our evolution-based method is not ideal for generating close variants with modified PAM specificity"? Please clarify or resolve the issue, currently, it seems like you offer modeling of variants to bind the same target, which is still valuable but this sentence in the introduction made me think you were addressing the same issue to this analysis.

While proposing different PAM was at first an objective, the study just showed results in designing variants with improved activity. We tried to make the introduction less confusing by removing this part.The paragraph that formerly contained this part was updated as follow:

“ We evaluate our strategy on the design of Cas9 variants. The Cas9 protein is an RNA guided nuclease from the prokaryotic immune system known as CRISPR-Cas9 \cite{doudna2014}. It forms a complex with a guide RNA, which directs Cas9 to bind complementary DNA sequences and introduce a double strand break. The first step in target recognition involves binding to a small DNA sequence motif found immediately downstream of the target sequence and known as the Protospacer Adjacent Motif (PAM). This interaction is mediated by a specific domain of the Cas9 protein, the PAM interacting domain (PID). In the case of the well described Cas9 protein from Streptococcus pyogenes (SpyCas9), the PAM motif is composed of any nucleotide followed by two guanines (NGG). Previous works showed that the PID of Cas9 can be swapped between different variants while retraining Cas9's functionality (\cite{nishimasu2014a,ma2019}), suggesting that the PID is relatively independent of the rest of the protein. In this study, we thus chose to model the PID only, without integrating the rest of the enzyme into our design efforts.”

By using the BuildModel command, you can isolate the effect of each mutation and obtain the relative DDG (free energy change) as the output. This will allow you to assess the impact of individual mutations. Additionally, you can separate the effects on stability from those on DNA interaction, if desired.

See reply to comment above

Minor comments:

- “studying protein interactions with ligands such as other proteins, RNA, DNA, and metals.”
→ cite any method (suggestion: https://pubmed.ncbi.nlm.nih.gov/29608705/; https://pubmed.ncbi.nlm.nih.gov/31732673/ ;)

Done, thank you!

- “As we see, we reach a maximum for intermediate value of γ that outperforms standard RBM (γ = 0)”
→ state which γ value and explain better why it is significant.

This was corrected as follow:

“ As we see, we reach a maximum for intermediate value of γ that outperforms a standard RBM ($\gamma = 0$), demonstrating that supervision helps the RBM separate PIDs with different recognition motifs in the latent space. Our model also outperformed a classifier trained uniquely on the labeled sequence data (high values of γ), highlighting the contribution of unlabeled data to the determination of a useful latent representation.”

- “Our model also outperformed a classifier trained uniquely on the labeled sequence data (high values of γ)”
→ where can I see this? Reference it in the text.

This was corrected as follow:

“Our model also outperformed a classifier trained uniquely on the labeled sequence data (high values of γ), highlighting the contribution of unlabeled data to the determination of a useful latent representation.”

- Fig4.a
→ the caption of the figure should explain the figure. I couldn’t understand it. What are the 2 red trapeze for? Also if possible would be nice to improve the aesthetic a bit.

We tried to make the figure easier to understand by having a single representation on the representation space and the manifolds. (see manuscript or below). We also update the caption:

“The Constrained Langevin Dynamics in the representation space consists of two steps. The first step is a round of sampling to evaluate the gradient of the main criterion and the gradient of the control criterion through the expectation formula (see methods), the second is a random step following the direction drawn by these two gradients (orthogonal projection of the main direction vector regarding the control direction). A Brownian noise is added to create randomness and diversity in the samples.”

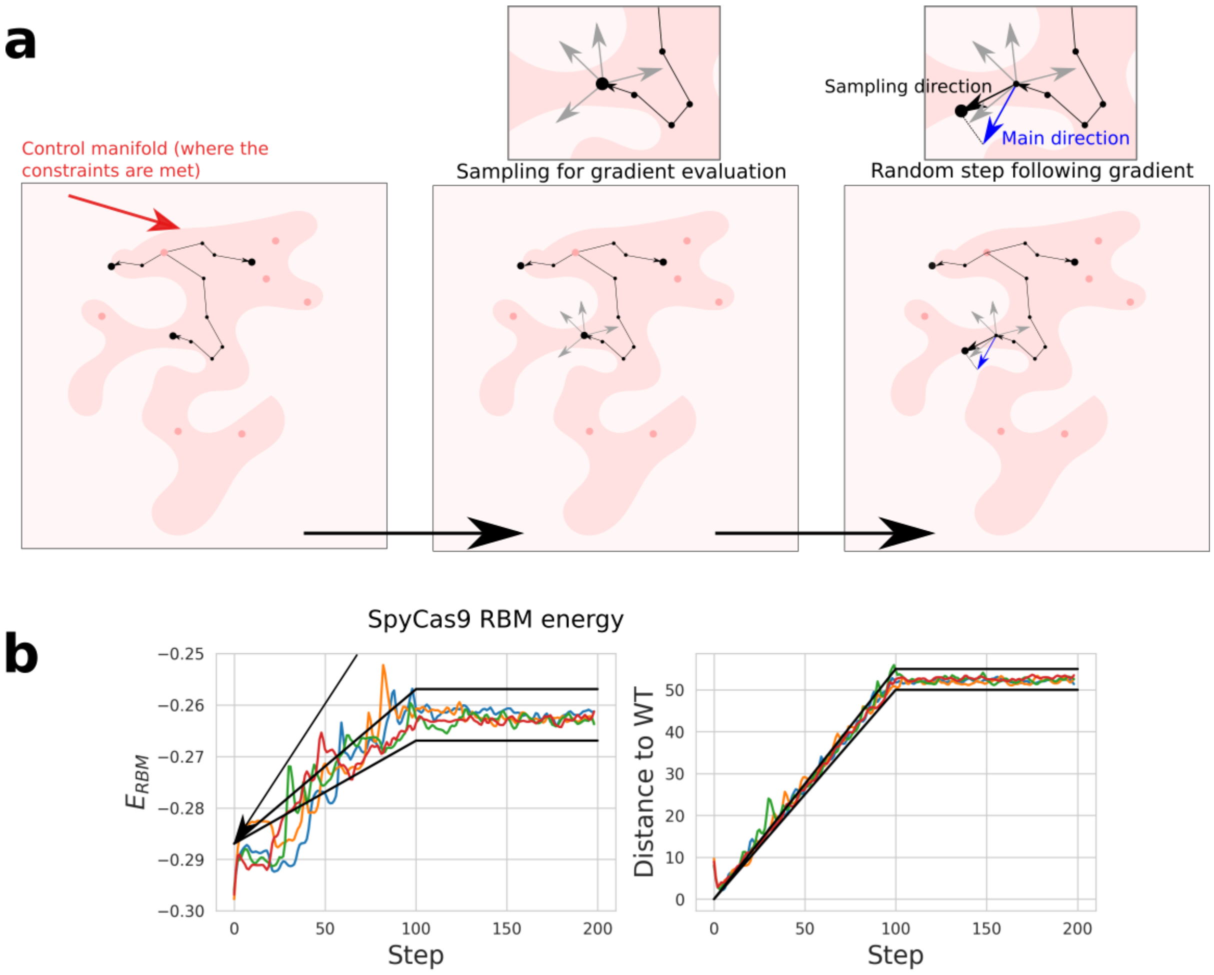

- “we show two examples of random walks with 5 different chains. We also display in supplementary (S5 file) some additional results on MNIST”
→ reference and provide an explanation of MNIST, and explain why it was useful to apply your approach to this specific dataset. The sentence in its current form lacks clarity and may lead to confusion.

We deleted MNIST from manuscript and supplementary as this felt a bit off subject. We wanted to display these results, so it could maybe inspire some future works with Langevin dynamics

- “We then replaced the amino acids by their values in the generated sequences”
→ instead of values, I would say ‘residue identities’.

We made the correction in the manuscript.

- “This results indicate that semi-supervised training on functional data, here the PAM recognition sequence, can improve the overall ability of the model to generate proteins that are likely to fold properly.”
→ these results indicate that this method produces sequences that are predicted to be stable variants of your protein according to FoldX. But to state this you have to repeat the analysis in the correct way explained above. Also you can also (optionally) show that your

method provides variants with affinity to the DNA using AnalyseComplex as explained above.

We have recomputed our scores as described above and we believe that our results are now consistent with the updated statement:

“We show the distribution of FoldX $\Delta \Delta \mathrm{G}$ values obtained for different values of γ in Fig~\ref{fig:ssl-rbm2}\textbf{b}. We observe a balanced regime where intermediate values of γ are better than in the regimes with weak (small values of γ) and with strong (high values of γ) feedbacks from the classifier. A peak is reached for values of γ around 5. This suggests that using semi-supervised training on functional data — specifically, the recognized PAM — enhances the model's capability to generate protein variants predicted to be stable by FoldX. This is promising for the creation of experimentally active sequences.”

- “These sequences were generated utilizing the representation space of a self-supervised learning Restricted Boltzmann Machine (SSL-RBM) ”
→ semi or self supervised?

“Semi”, it was corrected in the text

- “We selected 71 sequences spanning different predicted scores to be assessed experimentally (29 with method 1 and 42 with method 2).”
→ you selected those 71 based on what? Even if it was random, explain it.

We tried to explain a bit more how we selectd the sequences:

“We selected 71 sequences for experimental examination. Out of these, 29 were produced using method 1 and 42 with method 2. These sequences span a spectrum of values for metrics such as RBM energy, FoldX $\Delta\Delta G$, and AlphaFold RMSD. Our aim in selecting these sequences was to span a large range of values for these metrics in order to best evaluate which metric or combination of metric would best predict functionality DNA sequences encoding the desired proteins were synthesized and introduced in E. coli.”

- Fig.5a,b
→ add axis names

Done, thank you!

- “AlphaFold2 is a framework that uses modern neural networks to predict a distance map from a sequence and an alignment of the sequence homologs. This distance map is then used to build a 3-dimensional model of the protein structure through physically motivated methods.”
→ I think this sentence is not correct: what do you mean with ‘through physically motivated methods’? Just reference the method, it is way better. Also in the text you use AlphaFold and AlphaFold2, which one do you use? Make it uniform in the text.

-> We change AlphaFold to AlphaFold2 everywhere it was spotted in the text.

-> This paragraph was rephrased as follows, and the problematic sentence doesn't appear anymore.

"We further attempted to compare the information obtained by FoldX with the more recent protein structure prediction tool AlphaFold2 \cite{jumper2021a}. For each experimentally tested variant, we predicted a model of the structure (using ColabFold \cite{mirdita2022}). This model was then aligned with the AlphaFold2-predicted model for the wild-type protein using the TMscore program \cite{zhang2004}. From this alignment, we derived the root-mean-square deviation (RMSD) between the models. RMSD measures the root of the mean squared distance between the backbone atoms of the two protein models. We also collected from AlphaFold2 the average pLDDT score for each model, pLDDT is a per-residue confidence score indicating the reliability of the predicted position for each amino acid in the protein structure, which in the past has been shown to correlate well with variant activity. This score can be found in \nameref{file:s3}. We found that the RMSD (AUC = 0.810) performed better than the pLDDT (AUC = 0.668) to predict the activity of experimentally measured variants."

- "On Fig 8a, we display the correlation with the structural"
→ you meant 8b?

This was corrected in the text.

- "Interestingly, models seemed to capture complementary information, as can be seen in the failure of AlphaFold to spot inactive samples which are correctly predicted as active by the RBM and vice-versa."
→ how can I visualize this observation?

We have modified this paragraph as follow to make it easier for the reader to visualize this observation:

"Interestingly, models seemed to capture complementary information, as can be seen in the failure of AlphaFold to spot inactive samples which are correctly predicted as active by the RBM (red dots on the bottom-right part of Fig6d) and vice-versa (red dots on the top-left part of Fig6d)."

- "Second, this motif is recognized by our reference enzyme and by the large majority of close natural variants present in our dataset, as indicated by the comprehensive phylogenetic tree provided in supplementary (S6 file). Due to these limitations, our evolution-based method is not ideal for generating close variants with modified PAM specificity."
→ rephrase to make it more clear

This was rephrased as follows:

"Second, this motif is recognized by our reference enzyme (SpyCas9) as well as by the large majority of close natural variants present in our dataset, as seen in the phylogenetic tree provided in supplementary (\nameref{file:s6}). The lack of examples of close variants with

different PAM sequences in our dataset makes it difficult for our method to propose such variants.”

- “One promising strategy could involve employing Deep Learning frameworks like ProteinMPNN [60], or other platforms specifically designed to model binding interactions [61]. These frameworks could serve as viable alternatives to FoldX, offering enhanced flexibility and potentially more robust modeling capabilities. ”
→Proteinmpnn is a sequence generator for input structures, while FoldX is a force field that calculates DDG (free energy change) for structure models. If proteinmpnn generates sequence variants for a given structure, its function seems to be more similar to the RBM (Restricted Boltzmann Machine) presented in this manuscript rather than FoldX. Also could you clarify what you mean by "more flexibility and
robust modeling"? It would be beneficial to engage in a more in-depth discussion on future aspects or, if not possible, omit this part of discussion altogether.

These frameworks seem able to give an accurate enough estimate of various structural properties (binding energy, stability) faster than FoldX which could make them an interesting alternative to FoldX. Moreover, these methods are differentiable, which makes them well suited for an integration in our pipeline.

In the absence of an evaluation of these methods in our current work, we do not discuss this part anymore in the manuscript

Supp mat
→ provide detailed explanation for all tables, what are the columns?

We added description of the labels in the Excel file to ease understanding of the table

Description: Here we show the chimeras tested in sec 2.1 (protein sequence, origin, experimental results)

name: name of the natural Cas9 variant from which the PID was extracted
seq: amino-acid sequence
aligned_seq: amino-acid sequence with gaps to be aligned with PFAM16595 MSA
type: subfamily of the PID variant (see Collias et al. 2021)
pam: pam recognized according to Collias et al. 2021
identity: similarity to SpyCas9-PID
expected: expected activity based on the determined PAM (1 if expected active since NGG is supposed to be recognized)
norm_repression: measured experimental activity
norm_repression_std: standard deviation measured over the 3 experiments

Description: here we detail the variants whose activity was tested. The sequence, computational scores and experimental validation are displayed

id: name of the variant tested
batch: number of the batch, due to its complete failure and having been done through other methods, batch 1 was excluded from main

seq: amino-acid sequences
aligned_seq: amino-acid sequences with gaps to be aligned with PFAM16595 MSA
dna_seq: DNA sequence synthetized for cloning into E. coli
sim_cas9: # mutation to SpyCas9
best_sim: # mutation to closest natural variant collected
e_rbm: RBM energy (negative PLL + log(Z))
fx_ddg: DDG calculed with FoldX to assess protein stability
fx_interenergy: Interaction energy calculated with FoldX
af2_tmscore: TM-score between AF2 model of wild-type and the one for the variant (calculated through M-score software)
af2_rmsd: RMSD between AF2 model of wild-type and the one for the variant (calculated through M-score software)
af2_plddt: pLDDT of the AF2 model
af2_ptmscore: pTMscore of the AF2 model
norm_repression: measured experimental activity (approximated to 0 for batch as the experiment was not conducted to term)
norm_repression_std: standard deviation measured over the 3 experiments
functional: level of functionality to use in tasks
